# Leveraging limited data from wildlife monitoring in a conflict affected region in Venezuela

**DOI:** 10.1038/s41598-024-52133-0

**Published:** 2024-01-19

**Authors:** Izabela Stachowicz, José Rafael Ferrer-Paris, Ada Sánchez-Mercado

**Affiliations:** 1 Department of Geobotany and Plant Ecology, Faculty of Biology and Environmental Protection, University of Łódź, Banacha 1/3, 90-237 Lodz, Poland; 2 Laboratorio de Biología de Organismos, Centro de Ecología, Instituto Venezolano de Investigaciones Científicas, Apartado 20632, Caracas, 1020-A Venezuela; 3 School of Biological, Earth and Environmental Sciences, University of New South Wales, Kensington, NSW 2052 Australia; 4 UNSW Data Science Hub, University of New South Wales, Kensington, NSW 2052 Australia; 5 Ciencias Ambientales, Universidad Espíritu Santo, 092301 Samborondón, Ecuador

**Keywords:** Biodiversity, Conservation biology, Tropical ecology

## Abstract

Efficient monitoring of biodiversity-rich areas in conflict-affected areas with poor rule of law requires a combination of different analytical approaches to account for data biases and incompleteness. In the upland Amazon region of Venezuela, in Canaima National Park, we initiated biodiversity monitoring in 2015, but it was interrupted by the establishment of a large-scale mining development plan in 2016, compromising the temporal and geographical extent of monitoring and the security of researchers. We used a resource selection function model framework that considers imperfect detectability and supplemented detections from camera trap surveys with opportunistic off-camera records (including animal tracks and direct sighting) to (1) gain insight into the value of additional occurrence records to accurately predict wildlife resource use in the perturbated area (deforestation, fire, swidden agriculture, and human settlements vicinity), (2) when faced with security and budget constraints. Our approach maximized the use of available data and accounted for biases and data gaps. Adding data from poorly sampled areas had mixed results on estimates of resource use for restricted species, but improved predictions for widespread species. If budget or resources are limited, we recommend focusing on one location with both on-camera and off-camera records over two with cameras. Combining camera trap records with other field observations (28 mammals and 16 birds) allowed us to understand responses of 17 species to deforestation, 15 to fire, and 13 to swidden agriculture. Our study encourages the use of combinations of methods to support conservation in high-biodiversity sites, where access is restricted, researchers are vulnerable, and unequal sampling efforts exist.

## Introduction

Biodiversity monitoring is essential to inform conservation decisions and actions^1^. However, monitoring and conservation efforts may be impeded and interrupted in tropical high biodiversity regions facing economic challenges, social crises, or military conflicts^2^, resulting in gaps in biodiversity data and affecting management and stakeholders’ decision-making processes^3^. Poorly defined conservation goals may accelerate deforestation in conflict and conflict -prone areas^2,4^. Although such challenges can develop quickly, their negative effects on conservation achievements can be long-lasting. In this study, our primary focus was on sampling and data collection within conflict-affected regions. However, it's essential to acknowledge that other crucial aspects within conflict, conflict- affected, and high-risk areas, such as the escalating threats to species and ecosystems, the decision-making processes surrounding conservation priorities, and the effective implementation of conservation actions, have not received an adequate level of scrutiny and assessment^5^.

Rapid and drastic changes in deforestation patterns make long-term monitoring more important for future decision making, however fieldwork conditions are extremely challenging. For example, large-scale vertebrate monitoring projects require numerous camera traps, regular inspections, maintenance, and technical interventions by field staff, activities that are vulnerable in high-risk areas. Many conflict zones are located in the tropics, where high ecological and cultural diversity remain undersampled due to logistic limitations (cost of deployment), security, or low efficiency (few records per sampling effort)^6^.

The primary threats to tropical forests are conversion to nonforest for agriculture, cattle ranching and mining, degradation of remaining forest through hunting (defaunation), selective logging, fire, fragmentation, and associated edge effects^7,8^. Deforestation in the tropical region has been associated with a combination of several intersecting factors, including economic, demographic, institutional, and policy, but the role of these factors could vary across the spatial and temporal scale. As examples, during the conflicts in the Democratic Republic of Congo, Liberia, Myanmar, and Nepal political actors may have diminished conservation actions contributing to deteriorating conditions in specific areas^9^. In Colombia deforestation in protected areas has increased during the post-conflict period^10^. In Venezuela, a megadiverse country, the ecological consequences of the ongoing socioeconomic crisis are multiple and remain largely unquantified.

**Figure 1:**
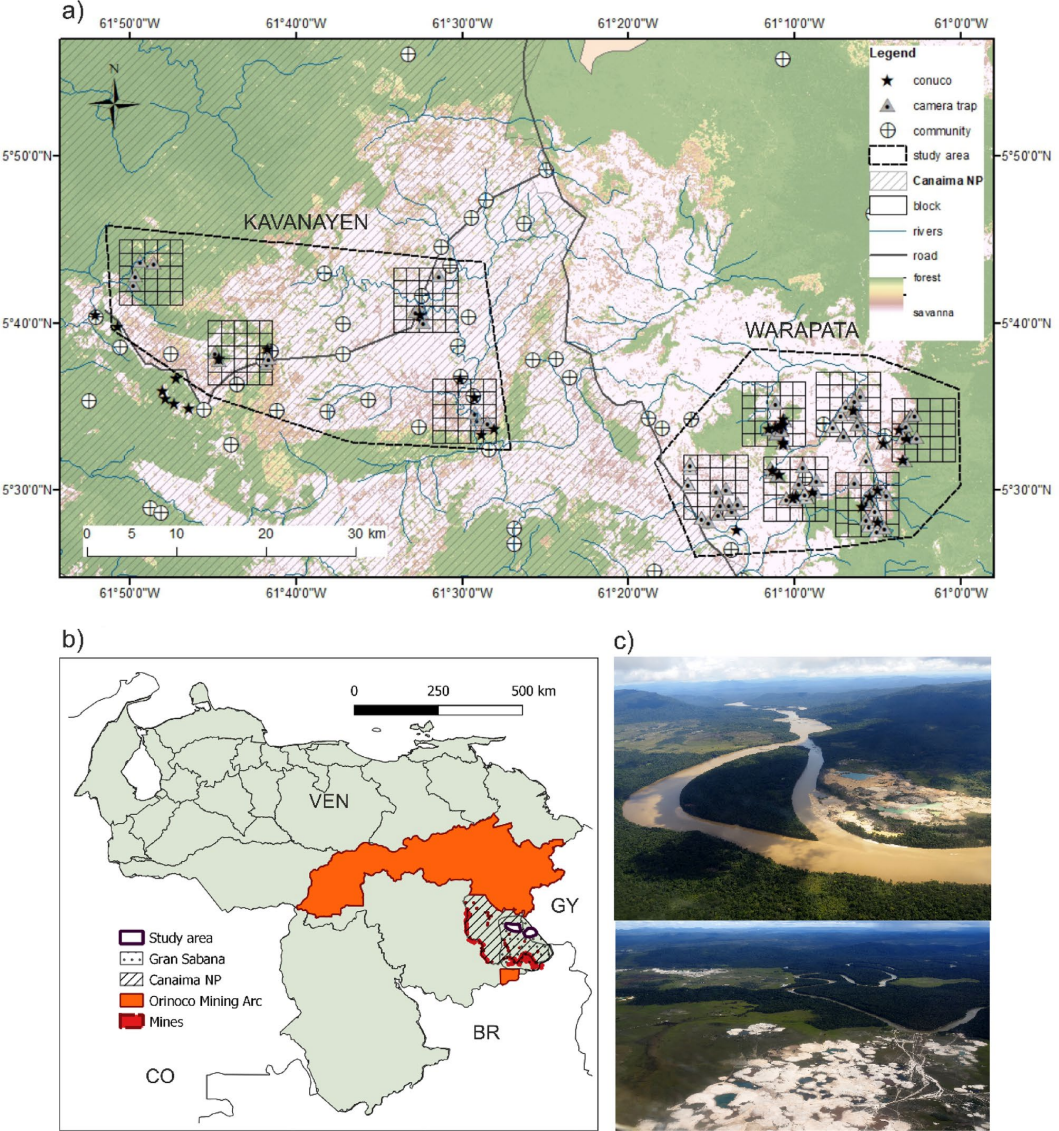
(**a**) Study area in Gran Sabana, Venezuela showing the location of the ten blocks surveyed with camera traps. (**b**) Orientation map showing the location of Orinoco Mining Arc, Canaima NP, Gran Sabana and the study area. Both maps were created using ArcGIS Desktop 10.8.2. software^22^. (**c**) Photos of illegal mining activity on the limit of Canaima National Park, Rio Caroni, up: 5.190031, −62.455521; down: 4.725977, −61.510104; author: Izabela Stachowicz, 2017.

In the Amazon, monitoring efforts are restricted to the lowlands^11–13^, while in the northern, upland areas, it is less known how landscape, cultural, and socioeconomic contexts shape the use of natural resources^14^. The Gran Sabana (GS) is a region located in the upland Amazon in southeastern Venezuela and belongs to the Guianan savanna bioregion (Fig. 1). This area is a complex upland landscape (450 to 2,810 m) of vast savannas with island-like patches of forest^15^. GS is proposed as a global conservation priority due to its high biodiversity and endemism of fauna and flora^16^. Seventy percent of GS area is protected within Canaima National Park (hereon Canaima), a UNESCO Natural World Heritage Site, with one of the highest deforestation rates in South America^17^ and, since 2017, considered to be of significant concern^18,19^. To date, only short-term monitoring of large and medium mammal species has been carried out in Canaima^20,21^.

In 2015, we initiated a monitoring program to evaluate species responses to different habitat perturbations at two locations on GS, Warapata and Kavanayen. We devised a strategy for annual camera trap surveys, employing a systematic approach that involved rotating locations throughout the study area over time. Our primary objective was to assess the impact of the disturbance on mammals applying multiseason occurrence models. Since 2016, the management of natural resources in the region has faced great challenges as the Venezuelan government has established a large-scale (111,843.70 km^2^, 12% of the country’s territory) mining development plan on the border of Canaima NP, known as the Orinoco Mining Arc (OMA)^23^. Mining operations originally intended to benefit the state in the deep economic crisis have bypassed any environmental impact evaluation, and more data is urgently needed to assess the ecological consequences of the OMA^24^. Conditions for monitoring and research are extremely unsafe due to the proliferation of uncontrolled mining activities, poor rule of law, and ongoing conflict between military forces and violent armed groups^25^. There is an increased health risk due to the reemergence of vector-borne diseases, such as malaria^26,27^, increased personal risk due to the activity of violent groups and violation of human rights^28,29^ and loss of critical partnerships with local Indigenous communities that been victims of multiple abuses, suffered radical changes in their livelihoods and even displaced from their original lands^30,31^.

**Figure 2:**
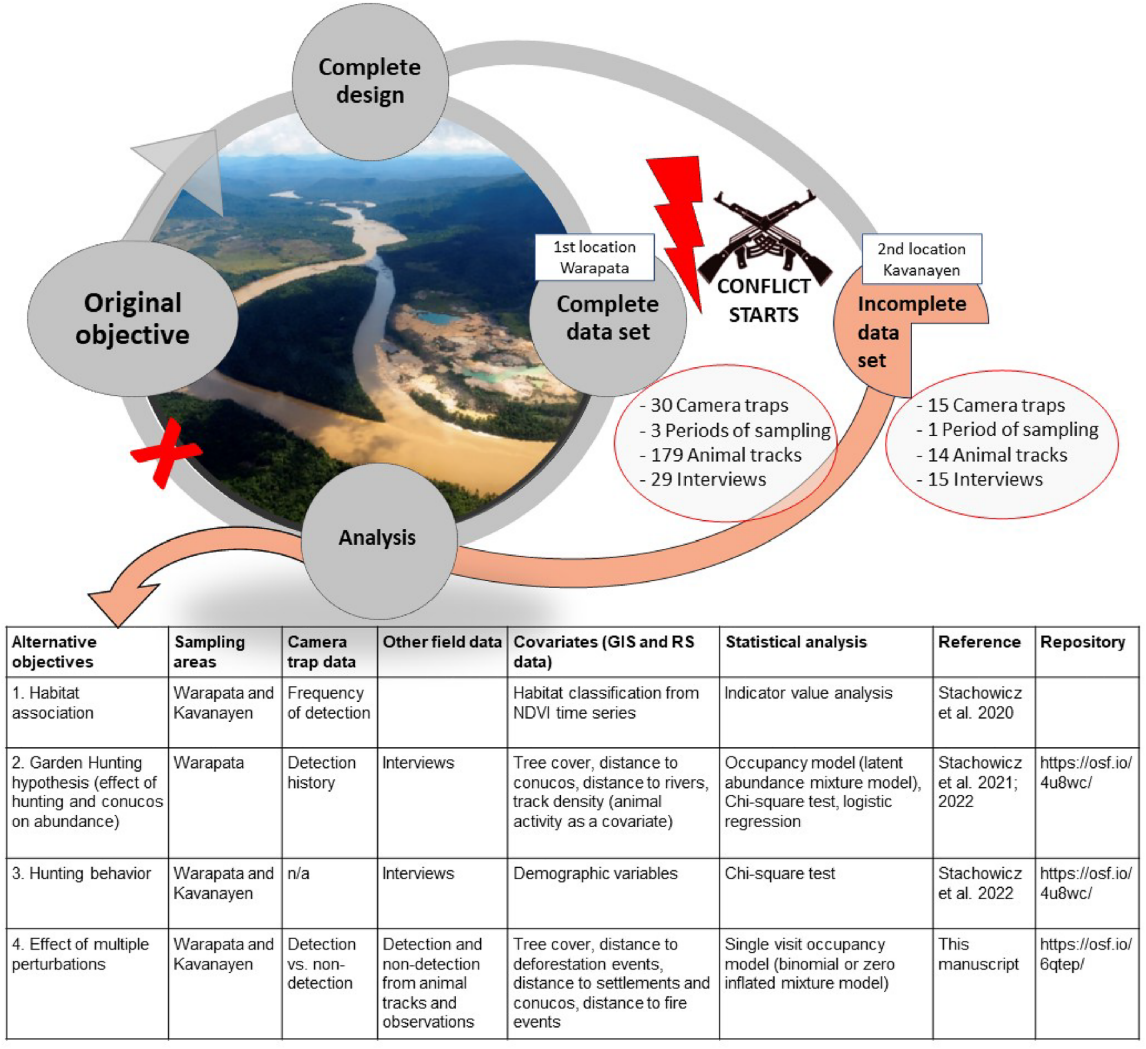
Conceptual model of interrupted monitoring faced in our study. Grey circles describe the steps initially planned for the study using the complete data set, while the orange circle describes the parallel course taken after the conflict that starts impeding the development of the study as planned, limiting sampling design and effort. The table shows evaluation of alternatives objectives that were evaluated and published. Photo of illegal mining activity on the limit of Canaima National Park, Rio Caroni, 5.190031, −62.455521, author: Izabela Stachowicz, 2017.

The deterioration of the situation affected our study area, located on the border with the OMA. Further, this situation had a spillover effect on funding agencies, which perceived Venezuela as too risky, thereby cutting off financial support. We completed our survey in the first locality (Warapata), but fieldwork in the second locality (Kavanayen) was interrupted when we experienced open distrust of local communities, the constant presence of the army and paramilitaries, and shortages of food and gasoline, rendering the originally planned temporal and geographical extent of our monitoring impossible. As a result, we were unable to complete fieldwork in Kavanayen, resulting in uneven coverage of sampling effort, and annual visits to the areas have been postponed indefinitely (Figs. 1, 2).

The raising of this conflict provided an opportunity to evaluate how we can use data from limited fieldwork to understand changes in biodiversity in a challenging conflict region. What strategies can we employ to maximize the utility of a robust design, taking into account the variations in spatial and temporal scales, while also assessing the existing evidence regarding species responses to various perturbation? Particularly, we were interested in comparing how resources selection models with imperfect detectability performed when we complement the data from our original camera trap survey with incidental observations (from off-camera sightings) or when we exclude data from poorly sampling regions and describe triangulation among data sources when facing security and budget constraints. Secondly, we use this framework (a) to explore the underlying patterns of medium-sized mammal and bird species responses to perturbations such as deforestation, fire, swidden agriculture (referred to as *conuco* herein) and proximity to human settlements, and (b) to compare whether the patterns, obtained with incomplete data, align with previous patterns based on more complete sampling. Here, we applied a resource selection model framework that accounts for imperfect detection via sampling effort covariates that relate to two sources of evidence. We compared model fits with different subsets of data to gain insight into the value of additional occurrence records (detections and nondetections) for estimating parameters and predicting of resource use.

Although our data is limited to a specific region and hence represent a case study from a conflict affected zone, we consider our approach illustrates how to maximize the use of fieldwork data combined with modeling to survey biodiversity in other countries with high biodiversity trapped in a human conflict zone.

## Results

### Evidence of species occurrence

Accumulated evidence of species occurrence comes from two complementary data streams: camera trap data and off-camera sightings (animal tracks and signs as well as direct observations) in two locations with different sampling effort, Warapata and Kavanayen (Fig. 1).

**Figure 3:**
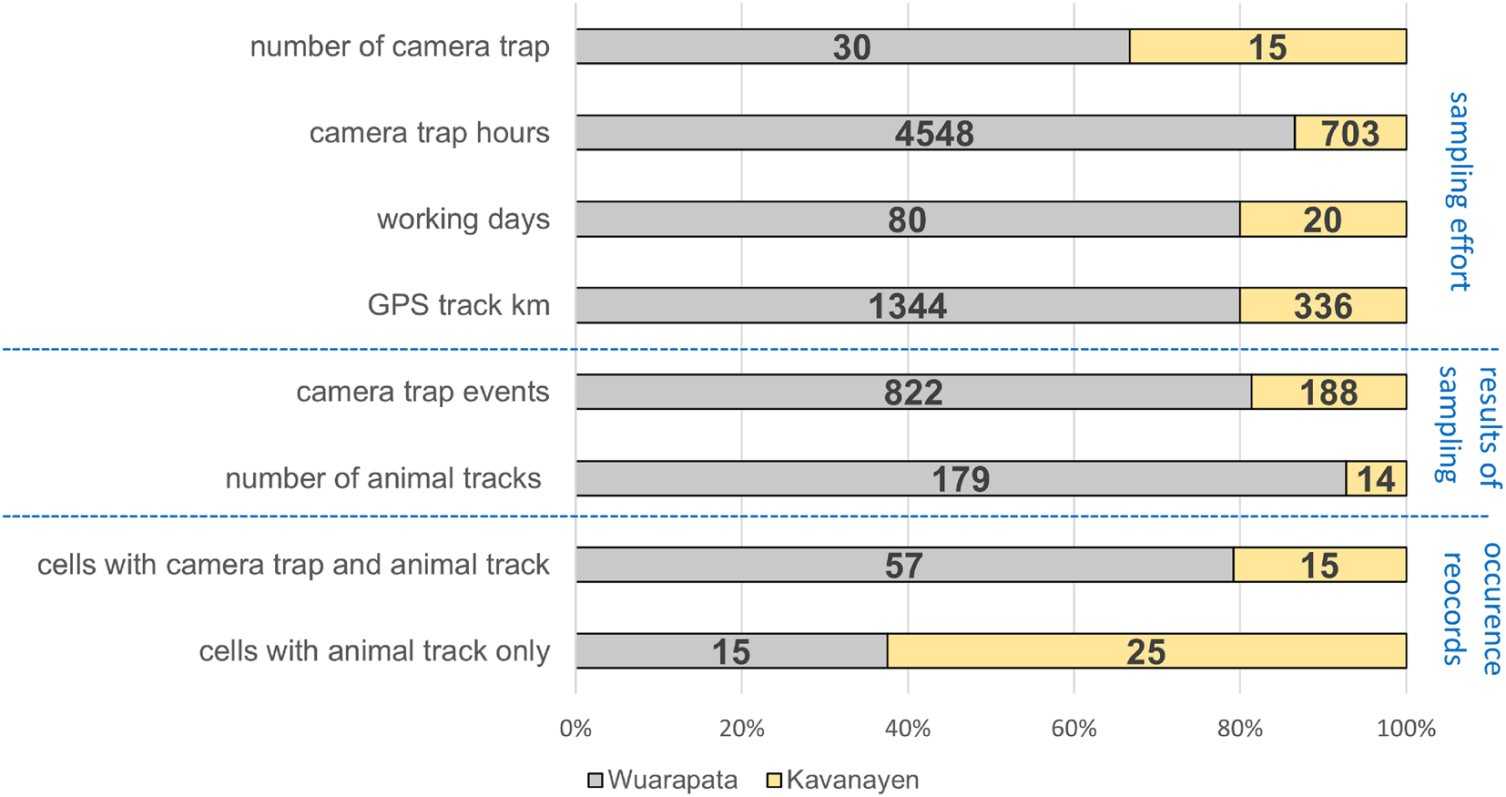
Comparation of sampling effort (number of camera traps, camera trap hours, working days, GPS track in km), sampling results (camera trap events and number of animal tracks and signs) and occurrence record per cell used in the resources selection model (cells with camera trap and animal track, cells with animal track only) between two localities, Warapata and Kavanayen. The locality of Warapata had a complete survey, while the survey in Kavanayen was incomplete.

For camera trap surveys, the location and duration of camera operation followed a stratified sampling design with larger blocks and smaller sampling units (grid cells; detailed description in Supplement 1). We consider Warapata to be well sampled with 4548 camera*days, 57 sampling units distributed among six sampling blocks, and a mean of 77.08 days of camera operation per grid cell. Kavanayen, on the other hand, was under-sampled with 703 camera*days, 14 sampling units distributed among four sampling blocks, and a mean of 50.21 days of camera operation per grid cell (Fig. 3).

Off-camera sightings of wildlife that we recorded incidentally along walking routes during the days of camera setup and maintenance. These records were mostly opportunistic since the routes were based on practical constraints (shortest or more accessible route), and the area covered and time spent in each sampling unit was very variable and not determined beforehand. However, evidence of species presence (direct observation, animal tracks, etc.) we documented rigorously, and a GPS log of the route was used to calculate the distance covered as a proxy of sampling effort. In Warapata, we visited 59 cells with a mean of 3.7 km walked in each cell, and in Kavanayen we visited 39 cells with a mean of 3.1 km walked in each cell (Fig. 3).

Species records for 28 species of mammals and birds (Supplement 1) were summarized as detections (values of one) and nondetections (values of zero) for a total of 112 sampling units across both locations, and sampling effort for cameras (camera*days) and field work (distance) are used as covariates to estimate the probability of detection.

### Model performance

For 19 species, models with spatial covariates (deforestation, fire, *conucos,* and proximity to human settlements) had strong data support compared to the null models (delta AIC > 2). For five species, the spatial covariate model could not be fitted or had a higher AIC than the null model. For the other four species, the results varied with different data sets with strong support for the spatial covariates when the full data set was used, but more support for the null model when using only camera trap data from a single region (Table Supplement 2).

When we compare best-fitting resource selection function models for each species (null or spatial covariate model) fitted using a minimal and a full input dataset, we observed obvious differences in predicted patterns of occurrence across the original study area (250 sampling units included in the original sampling design).

**Figure 4:**
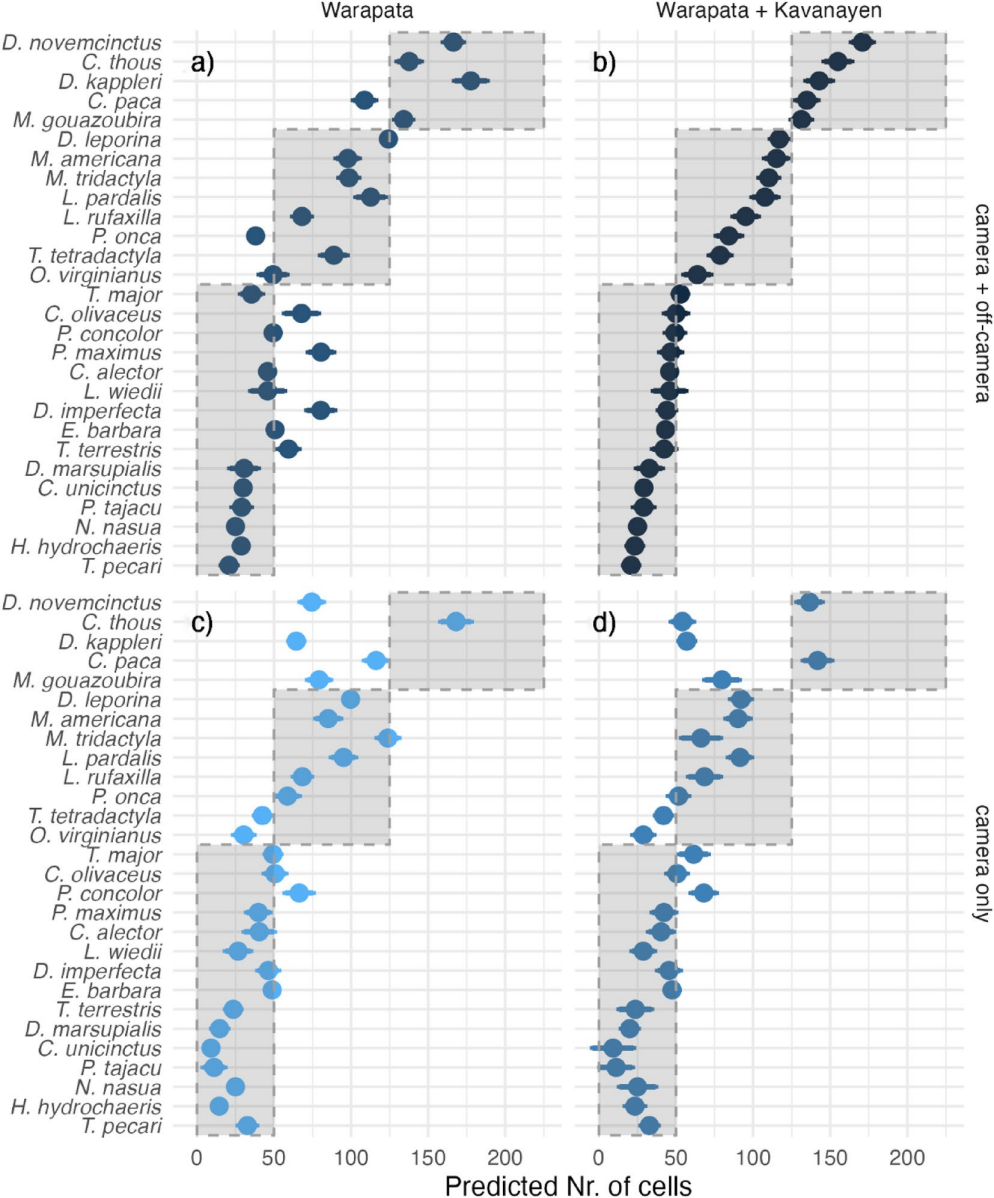
Predicted number of cells used by species in the study area using different combinations of data. (**a**) All available data in one sampling locations, (**b**) all available data, (**c**) camera trap data in one sampling location, and (**d**) camera trap data in both sampling sites.

A minimal dataset consists of only camera trap records from one region (57 cells with occurrence records, Fig. 4c), and the full input dataset includes all available data: camera trap records and off-camera sightings from both regions (72 + 40 cells, Fig. 4b). Arguably, we can expect the prediction from a minimal data set to be more biased due to the limited number of data records and underrepresentation of some combinations of covariates, while the prediction from the full input data set should be closer to the result that would be achieved with the original sample design.

Based on models fitted with all the available data, we could group species into three categories: (1) five widespread species predicted in more than 150 cells, (2) eight species with intermediate predictions (50 to 120 cells), and (3) the rest restricted to approximately 50 cells or less. The probability of resource use in the whole study area applying camera trap and observations was the highest for nine-banded armadillo (*D*. *novemcinctus*), crab-eating fox (*C. thous*), great long nosed armadillo (*D*. *kappleri*), lowland paca *(C. paca*) and gray brocket (*M*. *gouazoubira*) (Fig. 4b) and the lowest for collared peccary (*T. pecari*), capybara (*H. hydrochaeris*) and South American coati (*N. nasua*)*.*

The results from the model with the minimal data set have an intermediate level of agreement (Lin’s Concordance Correlation Coefficient of 0.74 with a confidence interval of 0.54–0.86) with much lower estimates of the conditional probability of use for widespread species, but predictions in the same range of values for intermediate and restricted species (Fig. 4c). The model fitted with data from camera records and off-camera sightings from the best sampled region predicted larger areas for four species with restricted ranges (Fig. 4a), but had the best agreement with the model based on all available data (CCC: 0.91, C.I. 0.82–0.96). Although the model using only on-camera records from both areas showed the least agreement (CCC: 0.67, C.I. 0.46–0.81) with underprediction for widespread and intermediate species (Fig. 4d).

We also expect that using all available data (both sources and both locations) will provide more robust estimates of parameters of the probability of use of resources for a larger number of species. There was more consistency between parameters estimates from cameras and observations in one or both regions (CCC > 0.58; Table 1). Using only cells with cameras, the number of adjusted models for species decreased for all perturbations measured, thus providing limited insights into the species’ response of covariates and much lower agreement (CCC < 0.40) with tree cover parameter estimates and complete disagreement with the other parameters (confidence interval overlaps with zero; Table 1).

**Table 1:** Lin’s concordance correlation coefficient (CCC) between the estimates of parameters for 25 species from the best spatial covariate models.

Parameter	Warapata camera and off-camera	Warapata + Kavanayen camera only	Warapata camera only
Tree cover	0.792 (0.589 to 0.901)	0.405 (0.0316 to 0.679)	0.383 (0.0162 to 0.659)
Deforestation	0.903 (0.765 to 0.962)	0.329 (−0.0820 to 0.645)	0.401 (−0.0949 to 0.737)
Fires	0.580 (0.250 to 0.790)	0.00581 (−0.448 to 0.458)	0.0987 (−0.348 to 0.509)
Communities	0.878 (0.677 to 0.957)	0.237 (−0.285 to 0.651)	0.137 (−0.333 to 0.552)
Conucos	0.737 (0.339 to 0.911)	0.309 (−0.221 to 0.698)	0.395 (−0.123 to 0.744)

Each column shows the CCC and 95% confidence intervals of the agreement between the reference estimates based on all the data (both regions with camera records and off-camera sightings) and the three subsets of data.

### Human resources and budget investment

**Figure 5:**
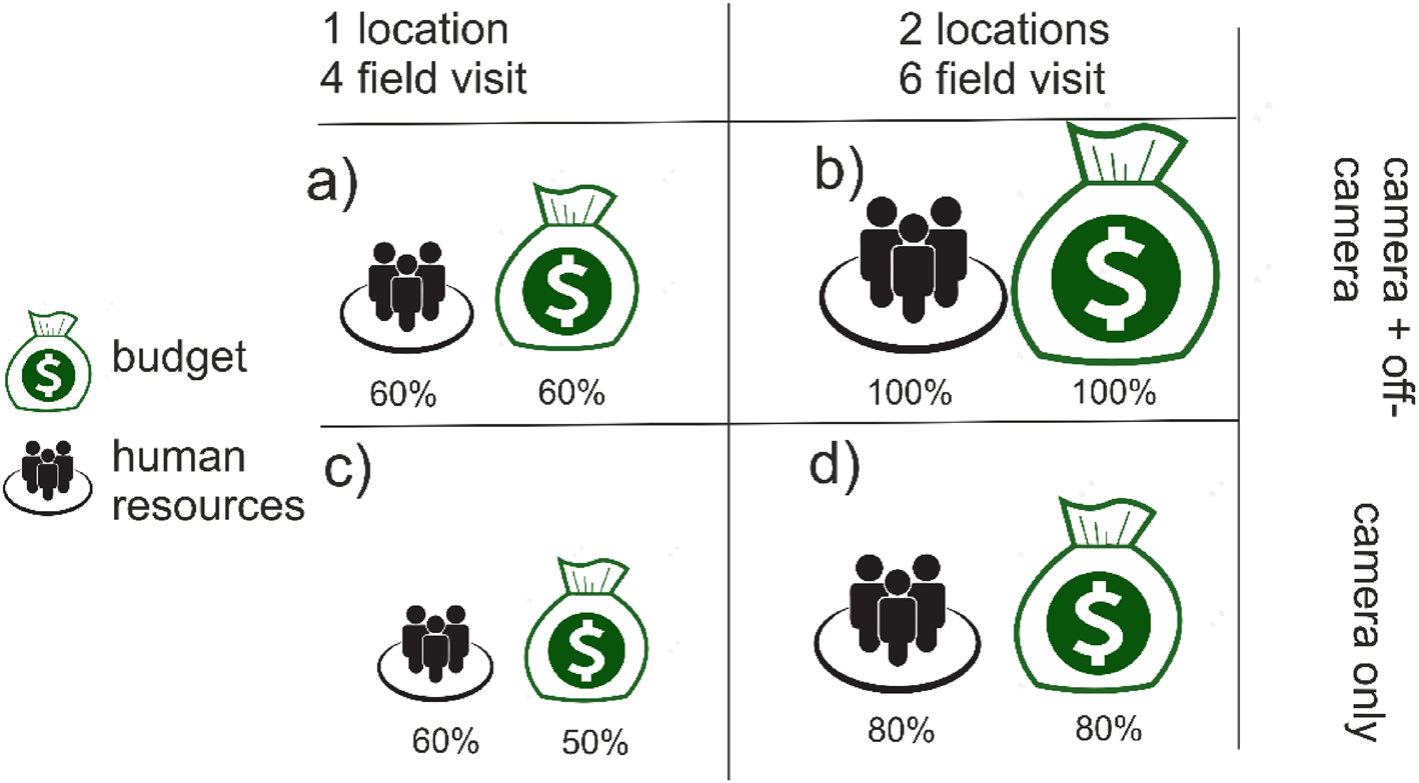
Budget and human resources investment across different combinations of sampling effort and number of locations sampled. (**a**) All available data in one sampling locality; (**b**) all the available data from two localities; (**c**) Camera trap data in one sampling locality; (**d**) Camera trap data in both sampling sites. We estimate costs based on transportation expenditures, as the number of visits varied across locations, and on the field team size, as salary and meals expenditure depends on days expend for visit and number of people (Supplement 3). We assumed that the equipment cost was similar across sampling localities. The maximum cost (100%) was reached when we used all available data (**b**). For the other combination of sampling effort, costs decreased, varying 50–80% of the maximum.

Human resource expenditure remained consistent regardless of whether the sampling effort involved solely cameras or a combination of cameras and direct observations at only one location (Fig. 5a, c). A small team of 2–4 people installed camera traps and recorded off-camera records. While expanding the study area, a noticeable shift occurred, leading to augmented budget allocation and increased human resource investment for sampling at two distinct locations (Fig. 5b, d). Interestingly, the model fit improved notably (Fig. 4a) when employing a combined approach of cameras and direct observations at a single location, in contrast to relying solely on cameras across two locations (Fig. 4d).

### Impact of perturbation on wildlife

**Figure 6:**
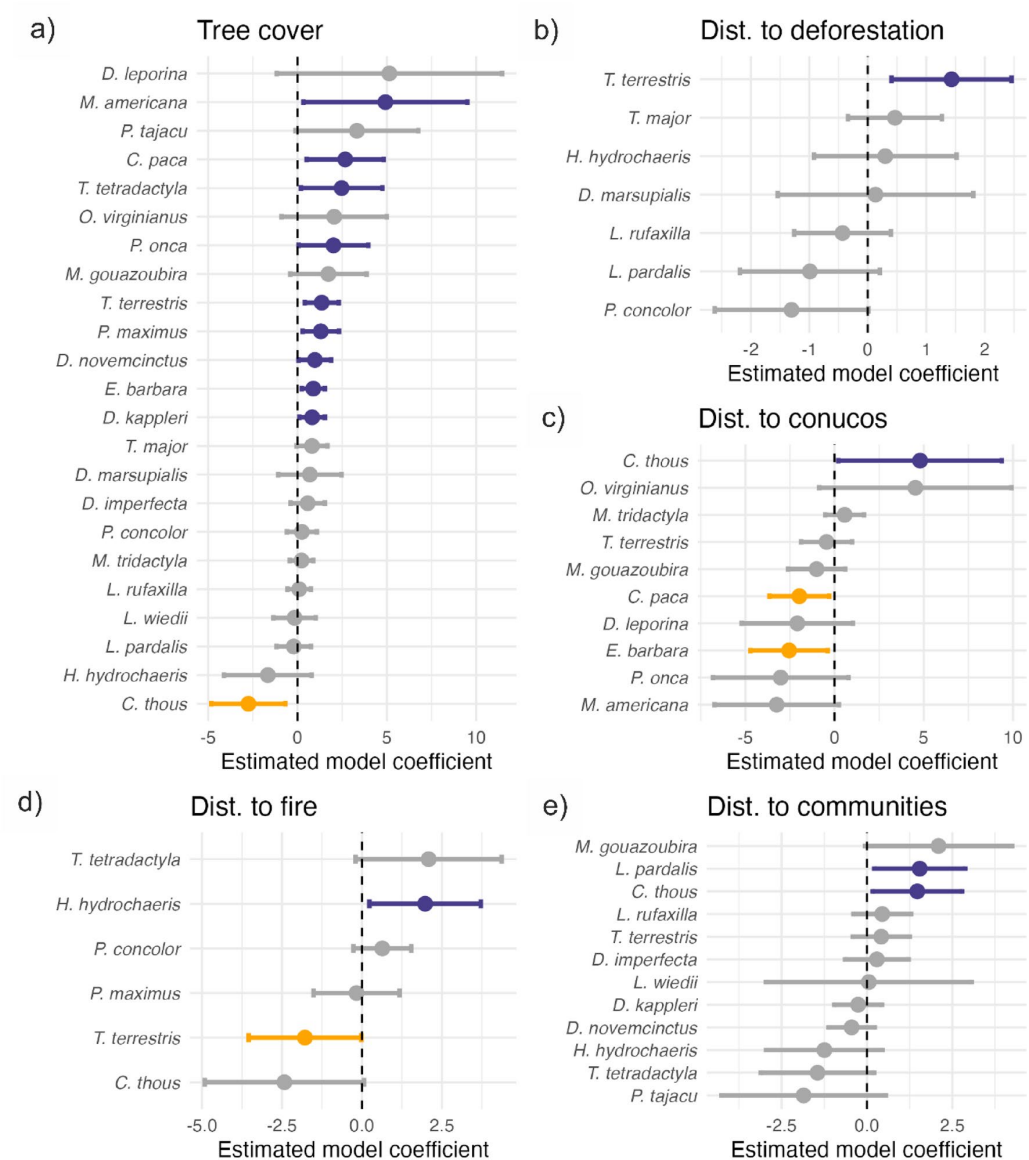
Resource selection function for the species with the best model selected by AIC using all available data. Coefficient values and their 95% confidence interval describing the relationship between species preferences and different variables are shown. (**a**) Percentage of forest cover, (**b**) distance to recent deforestation events, (**c**) distance to *conuco*, (**d**) distance to human settlements, and (**e**) distance to fire. Significant positive relationships are shown in blue, while significant negative relationships is in yellow. The grey dots and bars indicate no significant relationship.

Although some species had large positive or negative relationships with the covariates, only a few relationships could be considered significant (95% C.I. does not overlap zero; Fig. 6). The most common parameter fitted in the models was the association with forest (tree cover estimated from remote sensing). Using all available data (camera trap and off-camera sighting) from both sampling locations (Warapata and Kavanayen) we found that red brocket (*M. americana*), *C. paca,* South American tapir (*T. terrestris*), jaguar (*P. onca*), giant armadillo (*P. maximus*), nine-banded armadillo (*D. novemcinctus*), tayra (*E. barbara*), and greater long-nosed armadillo (*D. kappleri*) had positive relationships with tree cover, and *C. thous* had a negative relationship (Fig. 5a). Many of these relationships were also detected with data from one region only, but excluding data from observations led to mostly non-significant parameter estimates.

*T. terrestris* had a positive relationship with distance to deforestation events (avoid recently deforested areas) and puma (*P. concolor*) a strongly negative but nonsignificant relationship (prefers or is attracted to deforestation, Fig. 6b). Using camera trap data only, the model identifies positive relationships for white-tailed deer (*O. virginianus*) (Supplement 4).

Only one species avoided *conuco C. thous*, while *E. barbara* and *C. paca* had negative relationships with distance from *conucos* (prefers or is attracted to) (Fig. 6c). The magnitude of these relationships was not consistently detected with subsets of the data, but other species showed positive (southern tamandua—*T. tetradactyla* and *O. virginianus*) or negative (*P. onca*) relationships (Supplement 4).

For the effect of fire, we found that *T. terrestris* had a negative relationship (prefers or is attracted to, Fig. 6d).

Ocelot (*L. pardalis*) and C*. thous*, had positive relationships with distance to human settlements (avoid Pemón communities) when we applied all kinds of data (Fig. 6e). Using only camera traps did not demonstrate any significant relation (Supplement 4).

## Discussion

### Biodiversity monitoring challenges in conflict zones

Human conflicts generate complex social and environmental dynamics over space and time, with different effects on wildlife across regions and taxa. They often overlap with biodiversity hotspots^32–35^.

The dismantling of Venezuela’s environmental institutions and the collapse of its oil sector generated a chain reaction of unsustainable extraction of natural resource^36^. Illegal mining has increased drastically, along with the size of the human population and the influence of non-indigenous culture^24^. The most important environmental impacts of mining in the region are the destruction of ecosystems and rivers, the modification of topography, habitat fragmentation, and the pollution of water and fish by sediment and mercury^37–40^. More data is urgently needed to evaluate the ecological consequences of the OMA^24^.

In Venezuela, we witnessed the displacement of entire human communities due to the degradation of public health services and increased security risks, severing ties between our research group and local leaders, the near collapse of road infrastructure that increased the time and cost of travel in and out of the study region, and finally the robbery of camera trap equipment. This challenging conflict context, combined with budget limitations, resulted in limited sampling effort (eight months over three years). Consequently, important gaps in data acquisition occurred, decreasing sampling effort and escalating the challenge to data analyses and modeling for adequate data presentation.

Although the conflict interrupted our monitoring, our fieldwork still represents the baseline conditions before the disruption of large-scale socioeconomic changes in these communities. We have established a baseline methodology that can set future guidelines, considering sampling limitations, conservation opportunities, and increasing threats to biodiversity in this region. This study represents the first large-scale quantitative effort to sample the medium and large mammal fauna in the GS. We were able to detect 90% of expected medium and large mammals^41^ and propose stratified design that can be replicated successfully in other parts of GS or Venezuela and between mammals and other animals. During deciding how to organize fieldwork in conflict-affected area, the optimal approach in our case was to use two locations with camera trap and off-camera records. However, if budget constraints or limited human resources are a concern, it is more advisable to thoroughly cover one location with both on-camera and off-camera records rather than using cameras in two locations.

Unfortunately, these complex scenarios of fieldwork in conflict and conflict affected areas are faced by many researchers in other parts of the tropics^42^. The results of this study underline the importance of in-country research programs, local data acquisition, and offer an easy-to-apply modeling approach that seeks the maximal use of limited field data to answer crucial biodiversity questions about wildlife response to perturbation.

### Comparison of models addressing alternative objectives

The interruption of sampling effort presented us with the challenge of very heterogeneous datasets and the impossibility of fully completing the original analysis (Fig. 2). Initially, we used naive estimates of occupancy and frequency from camera trap data for both regions to study habitat preferences using indicator value analysis^20^, but this ignores the problem of imperfect detection. Our second approach was to use the detection histories from the best-sampled region to apply a Royal-Nichols occupancy model that links the probability of detection with the frequency of detections^21^. This analysis allowed us to analyze differences in occurrence and relative abundance of the species, but we had to discard all data from Kavanayen and limit the number of covariates to achieve acceptable goodness of fit. For the present study, we decided to combine camera trap data and field observations from both sampling regions to apply a single visit occupancy model^43^ that uses sampling effort to estimate the probability of detection. This approach uses all available evidence of habitat use over the duration of the study, but discards the additional information provided by detection histories. Due to the large sampling period and rotation of camera trap stations, we cannot assume closed populations during the entire duration of sampling, and therefore interpret the results of the Royal-Nichols and the single visit models as probabilities of resource use, rather than probability of occupancy^21^.

The resource selection model and the indicator value analysis agree on the importance of the forest as the most important habitat for native fauna. Half of the species indicated by both models displayed a similar preference for forest habitat^20^. Accounting for imperfect detection in the current analysis improves our ability to detect habitat preferences of species with restricted ranges, such as the endangered *P. maximus* and *T. terrestris* (Fig. 6a).

Our previous Warapata findings hint at the effect of *conucos* attracting certain species, while others seem to avoid them^21^. Our current analysis confirms the significant attraction of two species (*E. barbara* and *C. paca*), but does not detect significant avoidance by black curassow (*C. alector)*, while it suggests additional associations that were previously not detected or not statistically significant: detraction from conuco by *C.thous*, *O. virginatus,* and *T. tetradactyla* and attraction to conuco by *P. onca* (Fig. 5c, Supplement 4, Fig. 1c). The difference in both models can be illustrated with the example of *C. alector,* which had a large number of detection clustered in few camera sites. Multiple detections contribute more to the parameter estimation in the Royal-Nichols models and suggest high abundance far from *conucos*, while they are summarized as single presence records for the matching cells in the single-visit model and are thus not informative enough for detecting preferences. These mismatches between models emphasize the fact that although the single-visit model uses additional data, it still discards valuable information.

A complete and robust sample should always be the goal, as it will optimize the statistical power of the chosen analysis and improve data quality and model performance, but this turns out to be a factor beyond our control when working in conflict zones. The alternative analysis was chosen as the second-best approximation that uses the available evidence.

Recording incidental records was part of the original method and budget, thus we are adding useful detection records at a minimal extra cost (negligible extra time in the field). Sampling the second area for one period of time increased the cost by 40% (Figs. 5a, b., Supplement 3). The model performance showed a significant enhancement (Fig. 4a) when we integrated both cameras and direct observations at a single location. This stands in contrast to relying exclusively on cameras in two separate locations (as indicated in Fig. 4d). Based on our experience, it is more cost-effective to allocate time and human resources for both camera and off-camera recording in a single location than conducting a camera survey in two separate locations.

Currently, there are various models available, including integrated distribution models^44,45^, which also allow for the use of multiple data sources, but they can be challenging to implement with incomplete and unbalanced sampling.

### Perturbation in Gran Sabana

Upland Amazon ecosystems, such as this part of the Guiana Shield, face rapid changes in land use that affect the compositions of mammal and avifauna. For GS and Canaima, despite their importance as a UNESCO World Heritage Site and the long-standing presence of Pemón people, baseline knowledge about wildlife abundance patterns and how they change over time, space, and as a response to human-based stressors is limited. Our original objective was to understand the response of animals to perturbations related to the traditional livelihood of the Pemón: fires, deforestation related to swidden agriculture (*conucos*) and hunting (Fig. 1).

Off-camera records were an important complement to camera trap detections for predicting the proportion of the study region used by each species. The additional evidence of presence, leads to considerably larger predictions for a small group of species that would otherwise be considered of intermediate range (Fig. 4, top row vs. bottom row). Althougr data from the undersampled locality of Kavanayen contribute to additional presence records, it also contributes a larger proportion of nondetection records due to the limited sampling effort. Although the sampling effort covariate should help to account for imperfect detection, these additional nondetection records might misrepresent ranges for some species like, *T. terrestris*, or *P. maximus* (Fig. 4, top right vs. top left).

Taking into account these different contributions of data sources and regions, we would expect differences in the estimation of parameters of habitat association or the effect of perturbations. As above, the additional evidence of field observations leads to larger significant estimates for more species, while the models using only camera records seem nonconclusive in most cases (Fig. 6a–e, and Supplement 4). One possible reason is that the reduced number of records using camera traps alone has lower statistical power to detect weaker associations, resulting in models that failed to fit a larger number of parameters.

Some of the results are consistent with our previous analysis and other studies. Being a species ecologically related to forest, *T. terrestris* avoided the places where deforestation occurred (Fig. 6b). For this vulnerable and cryptic species, deforestation is of the most important threats^46^. Two species avoid human communities *L. pardalis* and *C. thous* and the last is the only one with a strong preference for the savanna ecosystem (Fig. 6a, d)^20^. The species attracted to *conuco* were *C. paca* and *E. barbara*, which is consistent with *The Garden Hunting hypothesis* (Fig. 6c,^21^). Surprisingly, a forest-preferring species, *T. terrestris*, was attracted to fire events (Fig. 6e). Detailed descriptions of each perturbation: deforestation, fire, *conuco* impact can be found in Supplement 3 and analysis of hunting impact can be found in^21,47^.

### Adaptive measures to mitigate challenges of fieldwork in the conflict region

Working in conflict zones poses significant challenges to researchers due to the volatile and unpredictable nature of these environments. Researchers often face risks to their personal safety, difficulties in accessing the population of interest, and limitations in the use of traditional research methods^48,49^. Based on our experience and review of the limited literature available, we suggest several adaptive measures and strategies to mitigate these challenges.

For example, seeking support and coordination from competent local authorities as well as collaborating with local organizations could provide insights into the region's dynamics, cultural nuances, and potential risks. This information should provide safety protocols to the field team^50^. In our case, our protocols include sampling in groups during safer hours, notifying authorities of sampling locations, and providing first aid training to all the team members. We have functional communication equipment to call authorities in an emergency (satellite phone, radio on long distance), carrying only essential equipment and minimizing displays of expensive equipment. Working in small teams is recommended, it reduced visibility, making logistics easier to manage, however, limits the scope of fieldwork. Further, risk assessments factors (e.g. safety risk index, remoteness) should be included as variables when defining sampling design to ensure that sampling is occurring in the most ecologically relevant areas but also in the most safe ones. Clearly, use of technology such as drone camera traps and remote sensing to enable remote research, data collection, and observation would reduce the need for physical presence in dangerous areas. The use of these technologies jointly with remote support systems to ensure data backup and transmission will allow researchers to navigate the challenges of conflict, conflict affected, and high-risk zones while conducting valuable research and contributing to positive outcomes.

## Conclusions

Here, we applied a resource selection model framework that accounts for imperfect detection via sampling effort covariates that relate to two sources of evidence. We compared model fits with different subsets of data to gain insight into the value of additional occurrence records (detections and nondetections) for estimating parameters and predicting of resource use.

Achieving cost-effictive monitoring in biodiversity-rich areas with ongoing and increasing social conflicts often requires combining different analytical approaches to account for data biases and incompleteness to make the most of available data. However, our understanding of species response to perturbations will be as incomplete as the data used, because the quality and amount of data determine the number of covariates that can be evaluated. However, a partial understanding is better than none. From our experience in Venezuela we learned that: (1) well documented field observations (meticulously recorded and georeferenced) are an important complement to camera trap records, recognizing the uncertainty of returning to a specific location; (2) different types of models allow an exploration of the relationship between variables and observations from different points of view and can be very informative to understand biases and data gaps; and (3) incomplete sampling effort can still contribute additional records even though these are of lower reliability. There is an urgent need to make even limited datasets from biodiversity hotspots available to the wider scientific community and the public, such as citizen science and biodiversity record platforms such as iNaturalist or GBIF^51^. With our study we would like to encourage research communities in high biodiversity in conflict, conflict affected, and high-risk zones to still report data even when collected with unequal sampling effort to help inform conservation action.

## Methods

### Study area

GS extends over 18,000 km^2^ in the Guiana Shield, or upland Amazon basin, and is characterized by a mosaic of tropical rainforest, pyric tussock savannas, and seasonally dry tropical shrublands within a complex landscape (500–1450 m elevation range)^16,52^. The Pemón people have inhabited GS for at least the last 500 years and currently, with 150 communities, are the main Indigenous People in GS (total population 30,146^14,53^).

They are based on slash and burn practices, which constitute the basis of shifting cultivation systems employing fire to create *conucos* in the forest interior^54,55^. Their low population density (0.93 habitant/km^2^^56^) was sustained by protein from fishing and hunting^57^, while *conucos* provide vegetables and tubers^58^. Typically, indigenous communities slash an area of < 1 ha of primary or secondary forest, which represents minimal pressure on the forest ecosystem^59^, with low levels of natural resource exploitation. However, in contrast to this, the last three decades have seen a rapid expansion of *conucos*, indigenous settlements, fires, and mining, which have become the main drivers of deforestation^15,21^.

The study area covers 1,442 km^2^ with elevation ranging from 700 to 1400 m above sea level within sector 5, to the north of GS, both inside and outside Canaima (Fig. 2). The vegetation in this area of the GS is characterized by shrubs dominated by *Clusia* spp. and *Gongylolepis* spp., broadleaf grasslands and savannas of *Axonopus* spp. interrupted by gallery forest patches and continuous evergreen montane forest near the Ilú-Tramén-tepui massif and Ptari tepui^16^. The climate is submesothermic ombrophilous, characterized by annual average temperatures between 18 and 24 °C and 2000–3000 mm of total annual rainfall with a weak dry season (< 60 mm/month) from December to March^14^.

### Fieldwork data and sampling collection

We established two sampling areas. The first (Warapata), sampled between September 2015 and April 2016, was close to the Venezuela-Guyana international border and was delimited by three Indigenous Pemón communities: Kawi (1100 m, 61.243 W 5.451 N), Uroy-Uaray (1093 m, 61.232 W 5.442 N) and Wuarapta (896 m, 61.157 W 5.512 N) (Fig. 2). We selected ten 50-km^2^ blocks (B01–B10) divided into 25 sampling units (cells) of 2 km^2^ each, covering the geographic and habitat diversity within the study area (Fig. 1). Blocks were selected to represent landscapes with different configurations of forest, savanna and shrub habitats, while cells were tentatively assigned to different classes based on remote sensing variables (tree cover and fire frequency) (detailed description of design can be found in Supplement 1). All maps were prepared using ArcGIS Desktop 10.8.2. software^22^.

In Warapata camera trap surveys were conducted between September 2015 and April 2016 for three 60-day periods. In each period, we placed the cameras (n = 30) following a stratified random sampling design that allowed us to cover 86 sampling units (details in^20^).

The second area (Kavanayen), sampled between May and July 2018, was located near the communities of Kavanayen (1222 m, 61.761 W 5.594 N), Liworibo (1255 m, 61.490 W 5.559 N) and the Parupa Research Station (1267 m, 61.544 W 5.5677 N). In Kavanayen, the camera trap survey was interrupted due to logistical limitations and the constant presence of the army and paramilitaries due to mining activities in OMA. This limited sampling to a 60-day period in May–July 2018 and 15 sampling units.

We recorded evidence of presence in two categories: (1) “on-camera” through camera trap surveys, (2) “off-camera” as opportunistic: direct observations and animal track record (scratches, caves, excrements and bones, details about sampling in^20^ and Supplement 1).

We mapped any track walked during fieldwork using a GPS (GPS track point records later used as an indicator of sampling effort) while direct observations were registered within 10–100 m of the trail, according to the visibility within each habitat type. Non-regular transect observation was applied. For each observation, we recorded the date, time, and geographic coordinates. We identified mammal species^60,61^ and birds^62^ using reference works for Venezuela and South America.

The challenging context of the fieldwork in southern Venezuela, combined with budget restrictions limited this work to six fieldwork trips, 10 blocks of sampling, 550 h of field sampling, and approximately 30 h of interviews with 41 hunters/local people. During the first fieldwork there was an assistant in the field, in the second there were two field assistants, in the rest of the fieldwork only the leader of the project participated with community members help. Crucial support was given by four people from the community who contributed (field work/translation/etc.) during the 100 days of field trips and visits to communities.

We opted to establish relative budget measures using a two-fold approach. First, we assessed the time allocation for on-camera and off-camera recording at each fieldwork site, Warapata and Kavanayen. Secondly, we systematically compared various fieldwork scenarios, factoring in human resources and budget allocations for both locations. Subsequently, these evaluations were juxtaposed with the projected cell usage of species. The outcome was presented as a percentage to gauge the comparative effort, with 100% serving as the baseline for recording both on-camera and off-camera in the two location.

### Measures of perturbations

We tested the influence of forest cover and distinct forest conversion drivers on the probability of use of each species. We used Global Forest Change products (^63^, version 1.7 updated in 2019) to estimate percentage tree cover and point location of detected deforestation events in the study area (1442 km^2^). GFC products use a time series of Landsat images with a spatial resolution of 30 m. Fire events for the study area were derived from a time series of Modis data^64^ 2011–2015 period for Warapata and 2013–2018 for Kayanayen (MODIS MCD14ML, Collection 6, pixel 1 km) with confidence > 40%. The conucos and human settlements were digitized from^65^ and additional *conuco* locations were taken during field work. For all four indicators of perturbance (deforestation event, fire event, *conucos* and human settlements) we calculated first distance surfaces in meters (euclidean distance to nearest point) and calculated their mean value over the area of the sampling units (cells). The hunting perturbance that was measured using interviews in two areas was described in detail in^21,47^.

### Resource selection model

We fitted a resource selection model to the aggregated on-camera and off-camera evidence of presence for 28 species of mammals and three species of birds identified in all sampling units visited.

We used a single-visit, zero-inflated binomial model with nonoverlapping covariates to account for imperfect probability of detection and use probability (i.e., resource selection or preference). This model allows combining different sources of evidence, provided that appropriate indicators of sampling effort are included^43^. The assumption of closed populations is not tenable with the available fieldwork data, thus we interpret the state variable as the probability of use rather than occupancy^66^.

We used the total number of working days as an indicator of sampling effort for camera traps and the number of GPS track point records as an indicator of sampling effort for other sources of evidence. The GPS track function was active during all field activities, so we considered this a reliable estimate of the time spent in each cell.

We tested the influence of forest cover and four distinct forest conversion drivers on the probability of use of each species. Therefore, we fitted a model with percentage forest cover, distance to recent deforestation events, distance to recent fire events, distance to *conucos*, and distance to human settlements.

Due to the inherent low information content in binary data, the estimation of parameters for binomial mixture models is difficult, and an exploration of alternative parameterizations is needed to stabilize estimators^67^. To aid this process, all variables were standardized to zero mean and unit standard deviation, and we tested alternative link functions for the probability of use (probit vs. complementary log–log), additive and multiplicative terms in the probability of detection, and stepwise reduction of terms.

We compared the resulting fitted model with a null model that assumes random mean probabilities of use for each sampling block (50 km^2^ blocks) but does not include any other spatial covariate. We compared the fitted and null models using the difference in AIC (delta AIC).

Using the best-fitting model for each species, we estimated the model parameters and their confidence interval to compare the response of the species to each of the covariates considered. We also used the best-fitting model to predict the probability of use per species for all 250 sampling units. The sum of these values represents the best estimate of the area that is used or occupied by each species, standard errors were estimated based on non-parametric bootstrap.

We repeated the model selection, parameter estimation and spatial prediction procedure four times: first with all available data (camera and observations) and all sampling units (Warapata + Kavanayen); and then using only subsets that excluded lesser quality data: using only camera trap data in both sampling units; using all available data in Warapata; and using only camera trap data in Warapata.

The study received permits from the Ministerio del Poder Popular para Ecosocialismo y Aguas 1419/3/33/2015 and Instituto Nacional de Parques (INPARQUES) 18/16 205, 156, 17 in Venezuela.

### Supplementary Information

Supplementary Information 1.

Supplementary Information 2.

Supplementary Information 3.

Supplementary Information 4.

## Data Availability

All datasets analyzed during the current study and code for reproducibility of the analysis are available in the Open Science Framework repository, https://osf.io/ey8ft/.
