# Supplementary material for: Leveraging limited data from wildlife monitoring in a conflict affected region in Venezuela
Source: Sci Rep. 2024 Jan 19;14:1673. doi: 10.1038/s41598-024-52133-0 (PMC10799001; doi:10.1038/s41598-024-52133-0)
Supplement: Supplementary file 1 — Supplementary Information 1. [file 41598_2024_52133_MOESM1_ESM.pdf]

Supplement 1 – Sampling and design description.

Leveraging limited data from wildlife monitoring in a conflict affected region in Venezuela

Izabela Stachowicz[1,2*], **José Rafael Ferrer-Paris**[3,4], **Ada Sánchez-Mercado**[3,5],

[1] Department of Geobotany and Plant Ecology, Faculty of Biology and Environmental Protection, University of Łódź, Banacha 1/3, 90-237 Łódź, Poland.

[2] Instituto Venezolano de Investigaciones Científicas, Centro de Ecología, Laboratorio de Biología de Organismos, Apartado 20632, Caracas 1020-A Venezuela.

[3] University of New South Wales, School of Biological, Earth and Environmental Sciences, NSW, Kensington 2052, Australia.

[4] University of New South Wales, UNSW Data Science Hub, NSW, Kensington 2052, Australia.

[5] Ciencias Ambientales, Universidad Espíritu Santo, Samborondón 092301, Ecuador

Sampling design for camera traps

In order to cover a large area and a diversity of habitats we selected ten sampling blocks (each covering an area of 50 km^2) across two regions (six in Warapata and four in Kavanayen). We considered forest cover, fragmentation and fire to be the most important factors shaping the GS landscape [1,2]. To systematically assess these factors, we employed a stratified design involving two distinct levels of analysis:

Block-level Analysis:

- For each of the ten blocks under consideration, we employed remote sensing data [3] to calculate the proportion of tree cover within each block.
- Fragmentation was quantified by determining the fractal dimension of the forest within each block [4].

Cell-level Analysis:

- Vegetation cover values were categorized into four levels for each cell: "continuous forest," "fragmented with predominance of forest," "fragmented with savanna predominance and savanna," following the approach by [3].
- Fire frequency over the past 14 years was assessed at three levels: "no fire," "one fire event in the last 14 years," and "more than one fire event in the last 14 years", considering the temporal limitation of MODIS data availability in 2015 [5].

Since only 30 cameras were available, we planned to sample one region at a time. The expected maximum sampling effort was 30 cameras deployed for 180 days of camera operation (5400

camera*days) in each region, with a maximum of 90 sampling units sampled in each region if all cameras are translocated in each period.

However, in order to maximize spatial coverage the sampling season was divided into three periods of 60-days each, and for each period a two-levels stratified random sampling was used to select 30 sampling units (with equal numbers per block) for camera deployment. This stratification ensures a balanced representation of sampling units with different coverage of habitat types and fragmentation in each block during each period. As a side effect of this, some sampling units with unique values within each block (e.g. sites with high tree cover within a block dominated by savanna) were selected for sampling in two or three periods and those cameras were neither relocated nor replaced. Minimal distance between cameras was 1 km.

Sampling effort for camera traps

We conducted one complete season of sampling in the first region (Warapata) with six blocks and five cameras per block for three periods of 60 days. Deviation from maximum sampling effort The rotation of sampling units per period yielded 57 sampled units instead of 90 due to many cameras kept for two or three periods in the same location, and 4548 camera*days mostly due to exhausted batteries or camera malfunction before the end of the period. This means that the cameras were operative for 84% of the planned time.

The second region was Kavanayen, where we selected four blocks for sampling, but sampling was conducted with a limited number of cameras, visits were delayed and ultimately sampling had to be suspended indefinitely due to security concerns. There were only 14 sampled units deployed for one period. Given the reduced amount of cameras available the expected maximum effort was 840 camera*days for one period, and we had 703 camera*days (84%) of camera operation.

Incidental off-camera sightings

Additional observations of wildlife were recorded with detailed field notes during all field visits related to camera deployment activities: once for camera set-up and installation, once at the end of each period for camera check-up, battery replacement and relocation, and then after the last period for camera deinstallation.

Walking routes were not standardized but followed the more convenient paths between camera locations as suggested by local field assistants and accessibility. A GPS log (GPS track function) was kept for each field visit.

We kept a record of all “off-camera sightings”, which can be based on direct observations (observations with visual identification of mammals and birds) or indirect evidence of mammal presence (animal tracks, scratches, trails, burrows, bones, and feces). Observations were opportunistic but documented rigorously with consistent level of detail. Each record included location (GPS waypoint), description of evidence (direct observation, animal tracks, scratches, etc), identification and qualitative indicator of certainty of identification.

Approximate sampling effort for off-camera sightings

Since the routes were not planned, we did not allocate sampling effort equally or consistently among sampling units. We calculated sampling effort *post-hoc* using the GPS log to calculate the distance covered within each sampling unit. This is an approximate measure of the effort invested in each unit, and we assume is proportional with time spent in the unit and the probability of observing wildlife directly or encountering indirect evidence.

We completed four field visits in Warapata (once for installation, two for camera set-up and relocation, and once for deinstallation). During these visits we walked 218 km in 59 sampling units spread across the six sampling blocks.

We completed two field visits in Kavanayen (once for installation and once for deinstallation). During these visits we walked 119 km in 39 sampling units across the four sampling blocks.

Results

In total, we installed cameras in 71 sampling units, achieving a total sampling effort of 5,251 camera*day. Sequences of photographs and videos were grouped in 7,569 events: 1,010 events with presence of mammals, 351 of birds and reptiles, and 6,082 empty frames or false positives (camera misfiring, likely due to vegetation movement or heat, among other reasons). We covered a distance of 338 km over 98 sampling units during the six field visits and recorded 165 off-camera sightings of 20 mammal species.

There was an important difference in sampling effort between regions and this reflects on the number of photographic events and off-camera sightings. Warapata contributes over 80% of the total number of photographic events and over 90% of off-camera sightings.

In both localities Warapata and Kavanayen, camera traps detected 28 species of mammals in eight orders, and 16 species of birds (Table S1): 25 mammal species in the well- sampled first location (Warapata) and 15 in the under-sampled second location (Kavanayen). However, three species were only detected in Kavanayen, two very rare (*S. venaticus* and *L. tigrinus*) and one common, but previously unrecorded for Gran Sabana (*M. pratti*; [6]).

Among the species with the highest values of frequency of detection index were lowland paca (*Cuniculus paca*), red-rumped agouti (*Dasyprocta leporina*), and crab-eating fox (*Cerdocyon thous*) (Supplement table 1). Species with the lowest values were margay (*Leopardus wiedii*), northern tiger cat (*Leopardus tigrinus*), collared peccary (*Pecari tajacu*), white-tailed deer (*Odocoileus virginianus*), southern naked-tailed armadillo (*Cabassous unicinctus*), common opossum (*Didelphis marsupialis*), and capybara (*Hydrochoeris hydrochaeris*). Among birds, the most common were grey-fronted dove (*Leptotila rufaxilla*) and black curassow (*Crax alector*). One of the rarest species detected was bush dog (*Speothus venaticus*), with one record.

Table A1. Nonvolant medium and large mammal and bird species recorded in the present study including its names in Arekuna (Pemón dialect), number of photographic events* (total number) and number of off-camera sightings**, status of red list following [7] and whether it was mentioned in interviews with local Pemón communities.

	Species	Species' name in Arekuna	Number of events*	Number of off-camera sightings**	Red list Venezuela
**MAMMALS**					
	**ARTIODACTYLA**				
	**Cervidae**				
Red Brocket	*Mazama americana* (Erxleben, 1777)	kutsari	32	10	**DD**
Gray Brocket	*Mazama gouazoubira* (G. Fischer, 1814)	kariyawku	69	18	**LC**
White-tailed Deer	*Odocoileus virginianus* (Zimmermann, 1780)	waikín	5	1	**LC**
	**Tayassuidae**				
Collared Peccary	*Pecari tajacu* (Linnaeus, 1758)	poyinke	4	2	**LC**
White-lipped Peccary	*Tayassu pecari* (Link, 1795)	pakirá	2	0	**VU**
	**CARNIVORA**				
	**Felidae**				
Margay	*Leopardus wiedii* (Schinz, 1821)	-	2		**VU**
Oncilla	*Leopardus tigrinus* (Schreber, 1775)	kaukau	1		**VU**

Ocelot	*Leopardus pardalis* (Linnaeus, 1758)	kaukan	24	3	**LC**
Jaguar	*Panthera onca* Linnaeus, 1758	temenen	27	4	**VU**
Puma	*Puma concolor* (Linnaeus, 1771)	kusariwara	32	7	**LC**
	**Canidae**				
Crab-eating Fox	*Cerdocyon thous* (Linnaeus, 1766)	maikan	94	37	**LC**
Bush dog	*Speothos venaticus* (Lund, 1842*)*	daiyai	1		**VU**
	**Mustelidae**				
Tayra	*Eira barbara* (Linnaeus, 1758)	yeruena	22		**LC**
South American Coati	*Nasua nasua* (Linnaeus, 1766)	kuachi	8		**LC**
	**CINGULATA**				
	**Dasypodidae**				
Greater Long-nosed Armadillo	*Dasypus kappleri* Krauss, 1862	-	56	18	**LC**
Nine-banded Armadillo	*Dasypus novemcinctus* Linnaeus, 1758	muruk	47	23	**LC**
Southern Naked-Tailed Armadillo	*Cabassous unicinctus* (Linnaeus, 1758)	-	4	2	**LC**

Giant Armadillo	*Priodontes maximus* (Kerr, 1792)	mauraimu	9	1	**EN**
	**PERISSODACTYLA**				
	**Tapiridae**				
South American Tapir	*Tapirus terrestris* (Linnaeus, 1758)	maikuri	35	21	**VU**
	**DIDELPHIMORPHIA**				
	**Didelphidae**				
Guianan White-eared Opossum	*Didelphis imperfecta* Mondolfi and Pérez-Hernandez, 1984	-	14		**LC**
Common Opossum	*Didelphis marsupialis* Linnaeus 1758	awaré	3		**LC**
	**PILOSA**				
	**Myrmecophagidae**				
Southern Tamandua	*Tamandua tetradactyla* (Linnaeus, 1758)	woiwo	6		**LC**
Giant Anteater	*Myrmecophaga tridactyla* Linnaeus, 1758	wareme	23	8	**VU**
	**RODENTIA**				
	**Cuniculidae**				
Lowland Paca	*Cuniculus paca* (Linnaeus, 1766)	uraná	343	7	**LC**
	**Dasyproctidae**				

Red-rumped Agouti	*Dasyprocta leporina* (Linnaeus, 1758)	akuri	236	4	**LC**
Green acouchi	*Myoprocta pratti* Pocok, 1913		2		**LC**
	**Hydrochoerinae**				
Capybara	*Hydrochoerus hydrochaeris* (Linnaeus, 1766)	parwena	10	7	**LC**
	**PRIMATES**				
	**Cebidae**				
Wedge-capped Capuchin	*Cebus olivaceus* Schomburgk, 1848	ibarakao	8		**LC**
	**Atelidae**				
Guyanan Red Howler	*Alouatta macconnelli* ^ Elliot, 1910	arauta			**LC**
**BIRDS**					

Arremon taciturnus		**8**	-	**LC**
Buteogallus meridionalis	woroiwo	1	-	**LC**
Cathartes aura	kurüm	2	-	**LC**
Crax alector	pauwi	48	-	**VU**
Crypturellus spp.		2	-	
Crypturellus variegatus		7	-	**LC**
Crypturellus soui	churima	11	-	**LC**
Geotrygon montana		1	-	**LC**
Leptotila rufaxilla	wakuma	68	-	**LC**
Mesembrinibis cayennensis		1	-	**LC**
Mimus gilvus	paraura	11	-	**LC**
Neomorphus rufipennis		1	-	**LC**
Penelope jacquacu	wora	8	-	**LC**
Tinamus major	marú	24	-	**NT**
Turdus albicollis		33	-	**LC**

* the event represents sequences of photos separated by less than 5 min and showing the same animal species and presumably the same individuals.

** the number of off-camera sightings reflects the total number of distinct evidence of all kinds of opportunistic direct observations and animal tracks (including scratches, caves, excrements, and bones) encountered during field visits.

^ species documented only by vocalization and interviews with local communities.

References

1. Huber O, Febres G, Arnal H. Ecological Guide to the Gran Sabana. Canaima National Park, Venezuela. Caracas, Venezuela: The Nature Conservancy; 2001.

2. Rull V, Montoya E, Nogué S, Vegas-Vilarrúbia T, Safont E. Ecological palaeoecology in the neotropical Gran Sabana region: Long-term records of vegetation dynamics as a basis for ecological hypothesis testing. Perspect Plant Ecol Evol Syst. 2013;15: 338–359. doi:10.1016/j.ppees.2013.07.004

3. Sexton JO, Song XP, Feng M, Noojipady P, Anand A, Huang C, et al. Global, 30-m resolution continuous fields of tree cover: Landsat-based rescaling of MODIS vegetation continuous

fields with lidar-based estimates of error. Int J Digit Earth. 2013;6: 427–448. doi:10.1080/17538947.2013.786146

4. Gneiting T, Ševčíková H, Percival DB. Estimators of fractal dimension: Assessing the roughness of time series and spatial data. Stat Sci. 2012;27: 247–277. doi:10.1214/11-STS370

5. Giglio L, Descloitres J, Justice CO, Kaufman YJ. An Enhanced Contextual Fire Detection Algorithm for MODIS. 2003;87: 273–282. doi:10.1016/S0034-4257(03)00184-6

6. Stachowicz I., Ferrer-Paris JR., Quiroga-Carmona M., Moran L., Lozano C. Baseline for monitoring and habitat use of medium to large non- volant mammals in Gran Sabana , Venezuela. Therya. 2020;11: 1–12. doi:10.12933/therya-20-891

7. Rodríguez JP, Garcia-Rawlins A, Rojas-Suárez F. Libro Rojo de la Fauna Venezolana. Caracas, Venezuela: Provita y Fundación Empresas Polar; 2015.
