# Supplementary material for: Leveraging limited data from wildlife monitoring in a conflict affected region in Venezuela
Source: Sci Rep. 2024 Jan 19;14:1673. doi: 10.1038/s41598-024-52133-0 (PMC10799001; doi:10.1038/s41598-024-52133-0)
Supplement: Supplementary file 2 — Supplementary Information 2. [file 41598_2024_52133_MOESM2_ESM.pdf]

Supplement to: Leveraging limited data from wildlife monitoring in a conflict affected region in Venezuela

Supplement 2 — AIC tables

Izabela Stachowicz   José R. Ferrer-Paris   Ada Y. Sánchez-Mercado

This supplementary documents includes four tables comparing null and spatial covariate models for each of the four combinations of input data (photographic records and off-camera sightings) and sampling regions (Warapata and Kavanayen).

Response variable y is the binary response with values of one (1) for detected and zero (0) for not detected. In all models a complementary log-log link function is used for the occurrence component and a logit link function is used for the detection component.

For each species the null model includes a fixed, spatially implicit effects of sampling block. The best spatial covariates model is shown when the difference in AICc (ΔAICc) is higher than zero.

Supplementary table A2: AIC table for models fitted using photographic records from Warapata as input. Response variable y is the binary response with values of one (1) for detected and zero (0) for not detected. Sample is limited to sampling units with cameras. The null model includes different occurrence probabilities for each sampling block and uses $cam = camera * days$ as covariate of detection probability. Occurrence covariates for the spatial covariate model are: bsq is tree cover; $dbsq$ is distance to nearest deforestation events; frs distance to nearest fire events; $dcom$ distance to nearest human communities or settlements; $dcon$ distance to nearest conucos. The spatial covariates models uses $cam = camera * days$ as covariate of detection probability. LL: Log Likehood, par: Number of parameters, n: number of observations, AICc: corrected Akaike Information Criterion, ΔAICc: difference between null and spatial covariates model.

	Null model			Spatial covariate model				
Species	Detect. covars.	LL (par, n)	AICc	Occurrence covariates	Detect. covars.	LL (par, n)	AICc	ΔAICc
C. alector	cam	-8.48 (8, 57)	35.96	—	—	—	—	—
C. olivaceus	cam	-5.55 (8, 57)	30.09	bsq + frs	cam	-5.07 (5, 57)	21.31	8.78
C. paca	cam	-24.82 (8, 57)	68.65	bsq + dcon	cam	-26.04 (5, 57)	63.27	5.38
C. thous	cam	-24.14 (8, 57)	67.29	bsq + dcon	cam	-24.80 (5, 57)	60.77	6.52
C. unicinctus	cam	-5.21 (8, 57)	29.42	—	—	—	—	—
D. imperfecta	cam	-10.72 (8, 57)	40.45	bsq + dcom	cam	-13.32 (5, 57)	37.81	2.64
D. kappleri	cam	-17.61 (8, 57)	54.22	bsq + dbsq	cam	-18.40 (5, 57)	47.98	6.24
D. leporina	cam	-14.27 (8, 57)	47.53	bsq + frs	cam	-17.36 (5, 57)	45.89	1.65
D. marsupialis	cam	-2.77 (8, 57)	24.55	bsq + frs	cam	-5.16 (5, 57)	21.49	3.06
D. novemcinctus	cam	-25.59 (8, 57)	70.17	bsq + frs	cam	-25.43 (5, 57)	62.03	8.14
E. barbara	cam	-17.73 (8, 57)	54.47	—	—	—	—	—
H. hydrochaeris	cam	-3.82 (8, 57)	26.64	bsq + dbsq	cam	-6.59 (5, 57)	24.35	2.28
L. pardalis	cam	-14.39 (8, 57)	47.78	bsq + dcom	cam	-16.13 (5, 57)	43.44	4.35
L. rufaxilla	cam	-18.49 (8, 57)	55.97	—	—	—	—	—
L. wiedii	cam	-4.03 (8, 57)	27.05	bsq + dcom	cam	-6.39 (5, 57)	23.96	3.10
M. americana	cam	-19.54 (8, 57)	58.07	bsq + frs	cam	-18.05 (5, 57)	47.27	10.80
M. gouazoubira	cam	-12.72 (8, 57)	44.44	bsq + dcon + frs + dbsq	cam	-9.19 (7, 57)	34.66	9.78
M. tridactyla	cam	-22.37 (8, 57)	63.73	bsq + frs	cam	-22.98 (5, 57)	57.13	6.60
N. nasua	cam	-3.29 (8, 57)	25.57	—	—	—	—	—
O. virginianus	cam	-6.69 (8, 57)	32.38	bsq + dbsq	cam	-7.96 (5, 57)	27.11	5.28
P. concolor	cam	-11.19 (8, 57)	41.38	bsq + dbsq	cam	-13.06 (5, 57)	37.30	4.08
P. maximus	cam	-10.42 (8, 57)	39.85	bsq + dcom	cam	-8.47 (5, 57)	28.11	11.74
P. onca	cam	-14.65 (8, 57)	48.30	—	—	—	—	—
P. tajacu	cam	-5.05 (8, 57)	29.10	—	—	—	—	—
T. major	cam	-12.98 (8, 57)	44.97	bsq + dbsq	cam	-15.75 (5, 57)	42.68	2.29
T. pecari	cam	-1.92 (8, 57)	22.84	bsq + dcom	cam	-4.17 (5, 57)	19.51	3.33
T. terrestris	cam	-10.13 (8, 57)	39.26	—	—	—	—	—
T. tetradactyla	cam	-9.84 (8, 57)	38.69	bsq + dcon + frs	cam	-11.03 (6, 57)	35.74	2.95

Supplementary table A3: AIC table for models fitted using photographic records from Warapata and Kavanayen as input. Response variable y is the binary response with values of one (1) for detected and zero (0) for not detected. Sample is limited to sampling units with cameras. The null model includes different occurrence probabilities for each sampling block. Occurrence covariates for the spatial covariate model are: bsq is tree cover; $dbsq$ is distance to nearest deforestation events; frs distance to nearest fire events; $dcom$ distance to nearest human communities or settlements; $dcon$ distance to nearest conucos. Covariates for the detection probability include $region$ and $cam = camera * days$. LL: Log Likehood, par: Number of parameters, n: number of observations, AICc: corrected Akaike Information Criterion, ΔAICc: difference between null and spatial covariates model.

	Null model			Spatial covariate model				
Species	Detect. covars.	LL (par, n)	AICc	Occurrence covariates	Detect. covars.	LL (par, n)	AICc	ΔAICc
C. alector	cam	-8.48 (12, 72)	46.25	—	—	—	—	—
C. olivaceus	cam	-5.55 (12, 72)	40.38	bsq + frs	cam	-5.07 (5, 72)	21.04	19.33
C. paca	cam	-33.49 (12, 72)	96.27	bsq + dcon + dbsq	cam	-34.82 (6, 72)	82.93	13.34
C. thous	cam	-24.14 (12, 72)	77.58	bsq + dbsq	cam	-27.47 (5, 72)	65.86	11.72
C. unicinctus	cam	-5.21 (12, 72)	39.70	—	—	—	—	—
D. imperfecta	cam	-10.72 (12, 72)	50.74	bsq + frs	cam	-13.45 (5, 72)	37.80	12.94
D. kappleri	region + cam	-20.00 (13, 72)	72.28	bsq + frs	region + cam	-21.42 (6, 72)	56.14	16.14
D. leporina	region + cam	-16.49 (13, 72)	65.26	bsq + dcon + frs	region + cam	-20.15 (7, 72)	56.05	9.21
D. marsupialis	cam	-2.77 (12, 72)	34.83	bsq + dbsq	cam	-5.41 (5, 72)	21.72	13.11
D. novemcinctus	region + cam	-27.84 (13, 72)	87.95	bsq + dbsq	region + cam	-29.12 (6, 72)	71.54	16.41
E. barbara	region + cam	-17.71 (13, 72)	67.70	bsq + dcon + frs + dbsq	region + cam	-17.95 (8, 72)	54.19	13.52
H. hydrochaeris	region + cam	-4.33 (13, 72)	40.94	bsq + dcom + dbsq	region + cam	-5.75 (7, 72)	27.26	13.68
L. pardalis	cam	-17.23 (12, 72)	63.75	bsq + dcom	cam	-19.11 (5, 72)	49.12	14.62
L. rufaxilla	cam	-18.49 (12, 72)	66.26	—	—	—	—	—
L. wiedii	cam	-4.03 (12, 72)	37.34	bsq + dcom	cam	-6.28 (5, 72)	23.47	13.87
M. americana	region + cam	-21.79 (13, 72)	75.85	bsq + frs	region + cam	-21.12 (6, 72)	55.54	20.31
M. gouazoubira	cam	-15.72 (12, 72)	60.72	—	—	—	—	—
M. tridactyla	cam	-22.37 (12, 72)	74.02	—	—	—	—	—
N. nasua	cam	-3.29 (12, 72)	35.86	—	—	—	—	—
O. virginianus	cam	-7.06 (12, 72)	43.41	bsq + dbsq	cam	-8.25 (5, 72)	27.41	15.99
P. concolor	cam	-11.19 (12, 72)	51.67	bsq + dbsq	cam	-13.40 (5, 72)	37.72	13.95
P. maximus	cam	-10.42 (12, 72)	50.14	bsq + dcom	cam	-9.48 (5, 72)	29.86	20.28
P. onca	region + cam	-16.90 (13, 72)	66.08	bsq + dcon + dcom	region + cam	-21.76 (7, 72)	59.27	6.80
P. tajacu	cam	-5.05 (12, 72)	39.39	—	—	—	—	—
T. major	cam	-12.98 (12, 72)	55.26	bsq + dcon + dbsq	cam	-15.84 (6, 72)	44.98	10.28
T. pecari	cam	-1.91 (12, 72)	33.11	bsq + dcom	cam	-4.17 (5, 72)	19.24	13.86
T. terrestris	cam	-10.13 (12, 72)	49.55	—	—	—	—	—
T. tetradactyla	region + cam	-9.84 (13, 72)	51.96	bsq + dcon	region + cam	-12.47 (6, 72)	38.23	13.73

Supplementary table A4: AIC table for models fitted using **photographic records** and **off-camera sightings** from **Warapata** as input. Response variable y is the binary response with values of one (1) for detected and zero (0) for not detected. Sample includes all sampling units visited during field work. The null model includes different occurrence probabilities for each sampling block. Occurrence covariates for the spatial covariate model are: *bsq* is tree cover; *dbsq* is distance to nearest deforestation events; frs distance to nearest fire events; *dcom* distance to nearest human communities or settlements; *dcon* distance to nearest conucos. Covariates for the detection probability are: *walk* is the distance walked in meters, and *cam* is the number of camera*days, either as additive (+) term or in interactions ($*$). LL: Log Likehood, par: Number of parameters, n: number of observations, AICc: corrected Akaike Information Criterion, ΔAICc: difference between null and spatial covariates model.

	Null model			Spatial covariate model				
Species	Detect. covars.	LL (par, n)	AICc	Occurrence covariates	Detect. covars.	LL (par, n)	AICc	ΔAICc
C. alector	walk + cam	-2.71 (9, 72)	26.32	—	—	—	—	—
C. olivaceus	walk + cam	-2.78 (9, 72)	26.45	bsq + frs	walk + cam	-3.01 (6, 72)	19.32	7.13
C. paca	walk * cam	-28.50 (10, 72)	80.61	bsq + dcon	walk * cam	-24.23 (7, 72)	64.22	16.39
C. thous	walk + cam	-37.20 (9, 72)	95.30	bsq + dcon + dcom + frs	walk + cam	-28.22 (8, 72)	74.72	20.58
C. unicinctus	walk * cam	-5.00 (10, 72)	33.62	—	—	—	—	—
D. imperfecta	walk * cam	-9.29 (10, 72)	42.20	bsq + frs	walk * cam	-10.62 (7, 72)	37.00	5.20
D. kappleri	walk * cam	-29.87 (10, 72)	83.34	bsq + dcon	walk * cam	-30.94 (7, 72)	77.63	5.71
D. leporina	walk + cam	-17.69 (9, 72)	56.28	bsq + frs	walk + cam	-9.27 (6, 72)	31.83	24.45
D. marsupialis	walk * cam	-2.77 (10, 72)	29.15	bsq + frs	walk * cam	-4.37 (7, 72)	24.50	4.65
D. novemcinctus	walk + cam	-26.88 (9, 72)	74.66	bsq + dcom	walk + cam	-29.12 (6, 72)	71.54	3.12
E. barbara	walk + cam	-16.99 (9, 72)	54.89	—	—	—	—	—
H. hydrochaeris	walk + cam	-9.65 (9, 72)	40.20	bsq + frs	walk + cam	-10.79 (6, 72)	34.88	5.32
L. pardalis	walk + cam	-19.27 (9, 72)	59.45	bsq + dcom + dbsq	walk + cam	-20.25 (7, 72)	56.25	3.20
L. rufaxilla	walk + cam	-18.27 (9, 72)	57.45	—	—	—	—	—
L. wiedii	walk * cam	-3.50 (10, 72)	30.60	bsq + dcom	walk * cam	-5.37 (7, 72)	26.49	4.11
M. americana	walk * cam	-21.87 (10, 72)	67.36	bsq + dcon	walk * cam	-20.55 (7, 72)	56.84	10.51
M. gouazoubira	walk * cam	-33.73 (10, 72)	91.07	bsq + dcom	walk * cam	-33.17 (7, 72)	82.08	8.99
M. tridactyla	walk + cam	-31.88 (9, 72)	84.66	bsq + dcon	walk + cam	-33.64 (6, 72)	80.56	4.09
N. nasua	walk * cam	-0.01 (10, 72)	23.62	—	—	—	—	—
O. virginianus	walk + cam	-7.80 (9, 72)	36.51	bsq + dcon	walk + cam	-6.87 (6, 72)	27.04	9.47
P. concolor	walk * cam	-15.60 (10, 72)	54.81	bsq + dbsq	walk * cam	-14.82 (7, 72)	45.40	9.42
P. maximus	walk + cam	-9.22 (9, 72)	39.35	bsq + dbsq	walk + cam	-12.51 (6, 72)	38.32	1.04
P. onca	walk + cam	-15.83 (9, 72)	52.57	—	—	—	—	—
P. tajacu	walk * cam	-7.84 (10, 72)	39.29	bsq + dcom	walk * cam	-5.11 (7, 72)	25.97	13.31
T. major	walk + cam	-12.66 (9, 72)	46.22	bsq + dcon + dbsq	walk + cam	-15.08 (7, 72)	45.90	0.32
T. pecari	walk + cam	-3.30 (9, 72)	27.50	—	—	—	—	—
T. terrestris	walk + cam	-16.24 (9, 72)	53.39	bsq + frs + dbsq	walk + cam	-14.22 (7, 72)	44.18	9.21
T. tetradactyla	walk * cam	-7.98 (10, 72)	39.57	bsq + dcon + dcom	walk * cam	-6.71 (8, 72)	31.70	7.87

Supplementary table A5: AIC table for models fitted using **photographic records** and **off-camera sightings** from **Warapata** and **Kavanayen** as input. Response variable y is the binary response with values of one (1) for detected and zero (0) for not detected. Sample includes all sampling units visited during field work. The null model includes different occurrence probabilities for each sampling block. Occurrence covariates for the spatial covariate model are: bsq is tree cover; $dbsq$ is distance to nearest deforestation events; frs distance to nearest fire events; $dcom$ distance to nearest human communities or settlements; $dcon$ distance to nearest conucos. Covariates for the detection probability are: $region$ (1 for Warapata, 0 for Kavanayen), $walk$ is the distance walked in meters, and cam is the number of camera*days, either as additive (+) term or in interactions (∗). LL: Log Likehood, par: Number of parameters, n: number of observations, AICc: corrected Akaike Information Criterion, ΔAICc: difference between null and spatial covariates model.

	Null model			Spatial covariate model			
Species	Detect. covars.	LL (par, n)	AICc	Covariates (occurrence | detection)	LL (par, n)	AICc	ΔAICc
C. alector	walk + cam	-2.71 (13, 112)	35.13	—	—	—	—
C. olivaceus	walk + cam	-2.78 (13, 112)	35.27	—	—	—	—
C. paca	region + (walk * cam)	-34.89 (15, 112)	104.77	bsq + dcon | region + (walk * cam)	-33.53 (8, 112)	84.47	20.30
C. thous	region + (walk * cam)	-45.72 (15, 112)	126.44	bsq + dcon + dcom + frs | region + (walk * cam)	-40.77 (10, 112)	103.72	22.72
C. unicinctus	walk * cam	-5.41 (14, 112)	43.14	—	—	—	—
D. imperfecta	region + walk + cam	-9.75 (14, 112)	51.83	bsq + dcom | region + walk + cam	-11.42 (7, 112)	37.92	13.91
D. kappleri	region + (walk * cam)	-32.43 (15, 112)	99.85	bsq + dcom | region + (walk * cam)	-34.74 (8, 112)	86.88	12.97
D. leporina	walk + cam	-21.17 (13, 112)	72.05	bsq + dcon | walk + cam	-14.86 (6, 112)	42.51	29.54
D. marsupialis	walk * cam	-2.77 (14, 112)	37.88	bsq + dbsq | walk * cam	-4.55 (7, 112)	24.17	13.70
D. novemcinctus	region + walk + cam	-30.21 (14, 112)	92.75	bsq + dcom | region + walk + cam	-37.43 (7, 112)	89.93	2.82
E. barbara	walk * cam	-26.36 (14, 112)	85.05	bsq + dcon | walk * cam	-25.44 (7, 112)	65.96	19.09
H. hydrochaeris	walk + cam	-9.64 (13, 112)	49.00	bsq + dcom + frs + dbsq | walk + cam	-10.38 (8, 112)	38.15	10.85
L. pardalis	region + walk + cam	-21.68 (14, 112)	75.68	bsq + dcom + dbsq | region + walk + cam	-23.20 (8, 112)	63.80	11.88
L. rufaxilla	walk * cam	-18.18 (14, 112)	68.69	bsq + dcom + dbsq | walk * cam	-23.44 (8, 112)	64.28	4.41
L. wiedii	walk * cam	-3.50 (14, 112)	39.33	bsq + dcom | walk * cam	-5.37 (7, 112)	25.83	13.50
M. americana	walk * cam	-25.61 (14, 112)	83.56	bsq + dcon | walk * cam	-26.49 (7, 112)	68.05	15.51
M. gouazoubira	region + walk + cam	-36.64 (14, 112)	105.62	bsq + dcon + dcom | region + walk + cam	-35.66 (8, 112)	88.72	16.90
M. tridactyla	region + (walk * cam)	-34.74 (15, 112)	104.48	bsq + dcon | region + (walk * cam)	-39.23 (8, 112)	95.87	8.61
N. nasua	walk * cam	-0.00 (14, 112)	32.33	—	—	—	—
O. virginianus	region + (walk * cam)	-9.28 (15, 112)	53.55	bsq + dcon | region + (walk * cam)	-5.62 (8, 112)	28.63	24.92
P. concolor	region + (walk * cam)	-13.93 (15, 112)	62.86	bsq + frs + dbsq | region + (walk * cam)	-13.91 (9, 112)	47.58	15.29
P. maximus	walk + cam	-9.22 (13, 112)	48.16	bsq + frs | walk + cam	-11.61 (6, 112)	36.01	12.15
P. onca	region + (walk * cam)	-21.98 (15, 112)	78.96	bsq + dcon | region + (walk * cam)	-24.29 (8, 112)	65.97	12.98
P. tajacu	region + (walk * cam)	-7.84 (15, 112)	50.68	bsq + dcom | region + (walk * cam)	-5.11 (8, 112)	27.62	23.06
T. major	walk + cam	-12.63 (13, 112)	54.97	bsq + dbsq | walk + cam	-17.19 (6, 112)	47.17	7.79
T. pecari	walk + cam	-3.30 (13, 112)	36.31	—	—	—	—
T. terrestris	region + walk + cam	-19.33 (14, 112)	70.99	bsq + dcon + dcom + frs + dbsq | region + walk + cam	-16.99 (10, 112)	56.17	14.82
T. tetradactyla	region + (walk * cam)	-7.18 (15, 112)	49.37	bsq + dcom + frs | region + (walk * cam)	-7.10 (9, 112)	33.96	15.41
