# Supplementary material for: Leveraging limited data from wildlife monitoring in a conflict affected region in Venezuela
Source: Sci Rep. 2024 Jan 19;14:1673. doi: 10.1038/s41598-024-52133-0 (PMC10799001; doi:10.1038/s41598-024-52133-0)
Supplement: Supplementary file 3 — Supplementary Information 3. [file 41598_2024_52133_MOESM3_ESM.pdf]

Supplement 3 – Budget and human resources investment.

Leveraging limited data from wildlife monitoring in a conflict affected region in Venezuela

Izabela Stachowicz[1,2*], José Rafael Ferrer-Paris[3,4], Ada Sánchez-Mercado[3,5]

[1] Department of Geobotany and Plant Ecology, Faculty of Biology and Environmental Protection, University of Łódź, Banacha 1/3, 90-237 Łódź, Poland.

[2] Instituto Venezolano de Investigaciones Científicas, Centro de Ecología, Laboratorio de Biología de Organismos, Apartado 20632, Caracas 1020-A Venezuela.

[3] University of New South Wales, School of Biological, Earth and Environmental Sciences, NSW, Kensington 2052, Australia.

[4] University of New South Wales, UNSW Data Science Hub, NSW, Kensington 2052, Australia.

[5] Ciencias Ambientales, Universidad Espíritu Santo, Samborondón 092301, Ecuador

Table A6. Contribution to different categories of fieldwork costs.

			Contribution to on-camera records in percentage	*Contribution to off-camera records*
Fixed costs	*cameras used for all sampling periods*	*45 cameras*	*100%*	*0%*
Field work Warapata	*Transport from and to sampling region*	*four visits*	*50%*	*50%*
	Interviews	*21hours*		*100%*
	walking between camera sites for installation, maintenance and deinstallation	*80 days*	*80%*	*20%*

Field work Kavanayen	*Transport from and to sampling region*	*two visits*	*50%*	*50%*
	Interviews	*9 hours*		*100%*
	walking between camera sites for installation, maintenance and deinstallation	*20 days*	*80%*	*20%*
