# Supplementary material for: Leveraging limited data from wildlife monitoring in a conflict affected region in Venezuela
Source: Sci Rep. 2024 Jan 19;14:1673. doi: 10.1038/s41598-024-52133-0 (PMC10799001; doi:10.1038/s41598-024-52133-0)
Supplement: Supplementary file 4 — Supplementary Information 4. [file 41598_2024_52133_MOESM4_ESM.pdf]

Supplement 4 – Perturbation description.

Leveraging limited data from wildlife monitoring in a conflict affected region in Venezuela

Izabela Stachowicz[1,2*], **José Rafael Ferrer-Paris**[3,4], **Ada Sánchez-Mercado**[3,5]

[1] Department of Geobotany and Plant Ecology, Faculty of Biology and Environmental Protection, University of Łódź, Banacha 1/3, 90-237 Łódź, Poland.

[2] Instituto Venezolano de Investigaciones Científicas, Centro de Ecología, Laboratorio de Biología de Organismos, Apartado 20632, Caracas 1020-A Venezuela.

[3] University of New South Wales, School of Biological, Earth and Environmental Sciences, NSW, Kensington 2052, Australia.

[4] University of New South Wales, UNSW Data Science Hub, NSW, Kensington 2052, Australia.

[5] Ciencias Ambientales, Universidad Espíritu Santo, Samborondón 092301, Ecuador

Figure. S1. Resource selection function for the species with best model selected by AIC using all the available data (top-right corner), camera trap data in both sampling sites (lower-right corner), camera trap data in one sampling locality (lower-left corner), and all available data in one sampling locality (top-left corner). Coefficient values and their 95% confidence interval describing relationship between species preferences and different variables are shown. a) percentage forest cover, b) distance to recent deforestation events, c) distance to conuco, d) distance to human settlements, e) distance to fire. Significant positive relationships are shown in blue, while significant negatives are in yellow. Grey dots and bars indicate no significant relationship.

a)

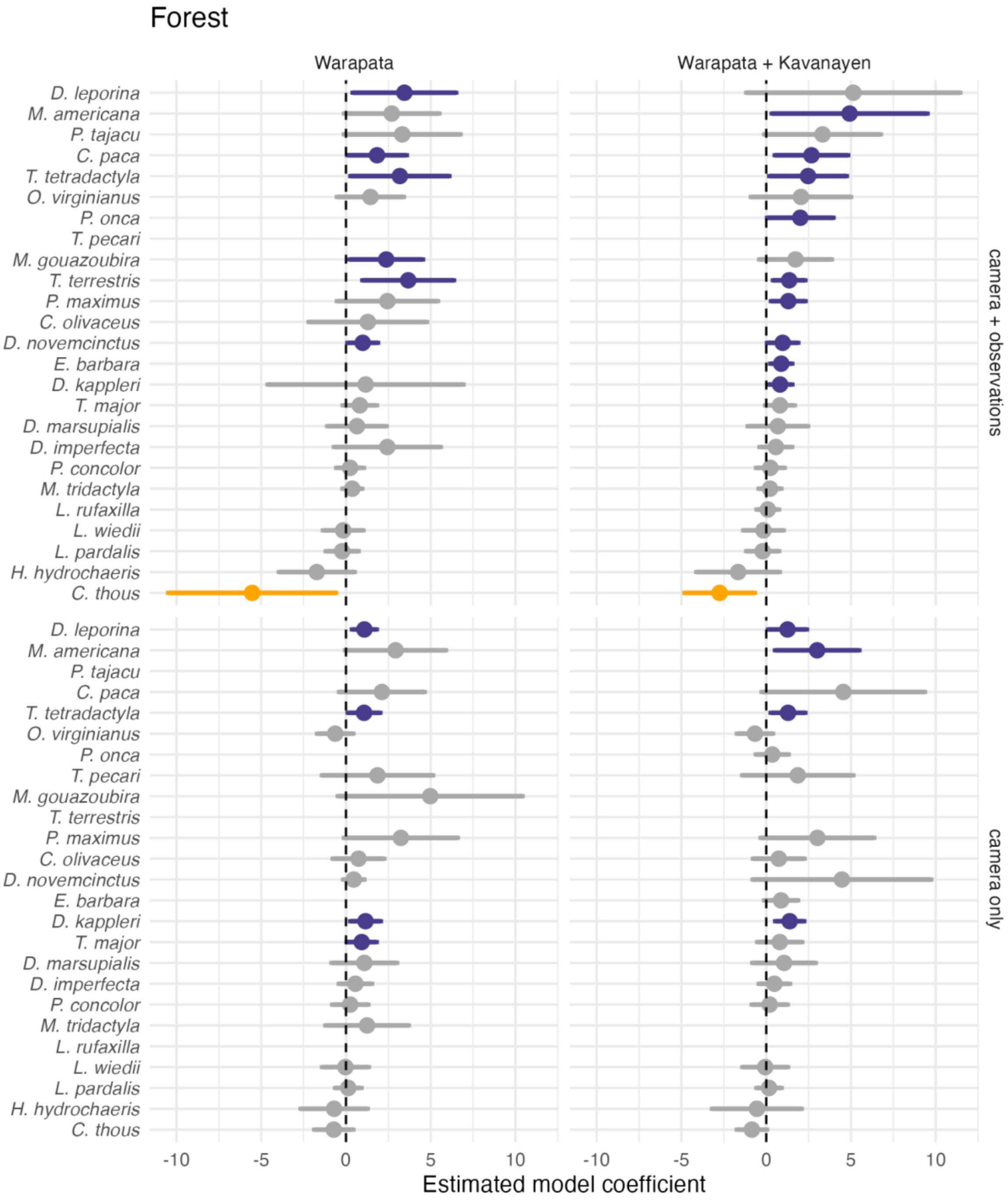
Forest
Warapata
Warapata + Kavanayen
D. leporina
M. americana
P. tajacu
C. paca
T. tetradactyla
O. virginianus
P. onca
T. pecari
M. gouazoubira
T. terrestris
P. maximus
C. olivaceus
D. novemcinctus
E. barbara
D. kappleri
T. major
D. marsupialis
D. imperfecta
P. concolor
M. tridactyla
L. rufaxilla
L. wiedii
L. pardalis
H. hydrochaeris
C. thous
camera + observations
camera only
-10
-5
0
5
10
Estimated model coefficient

b)

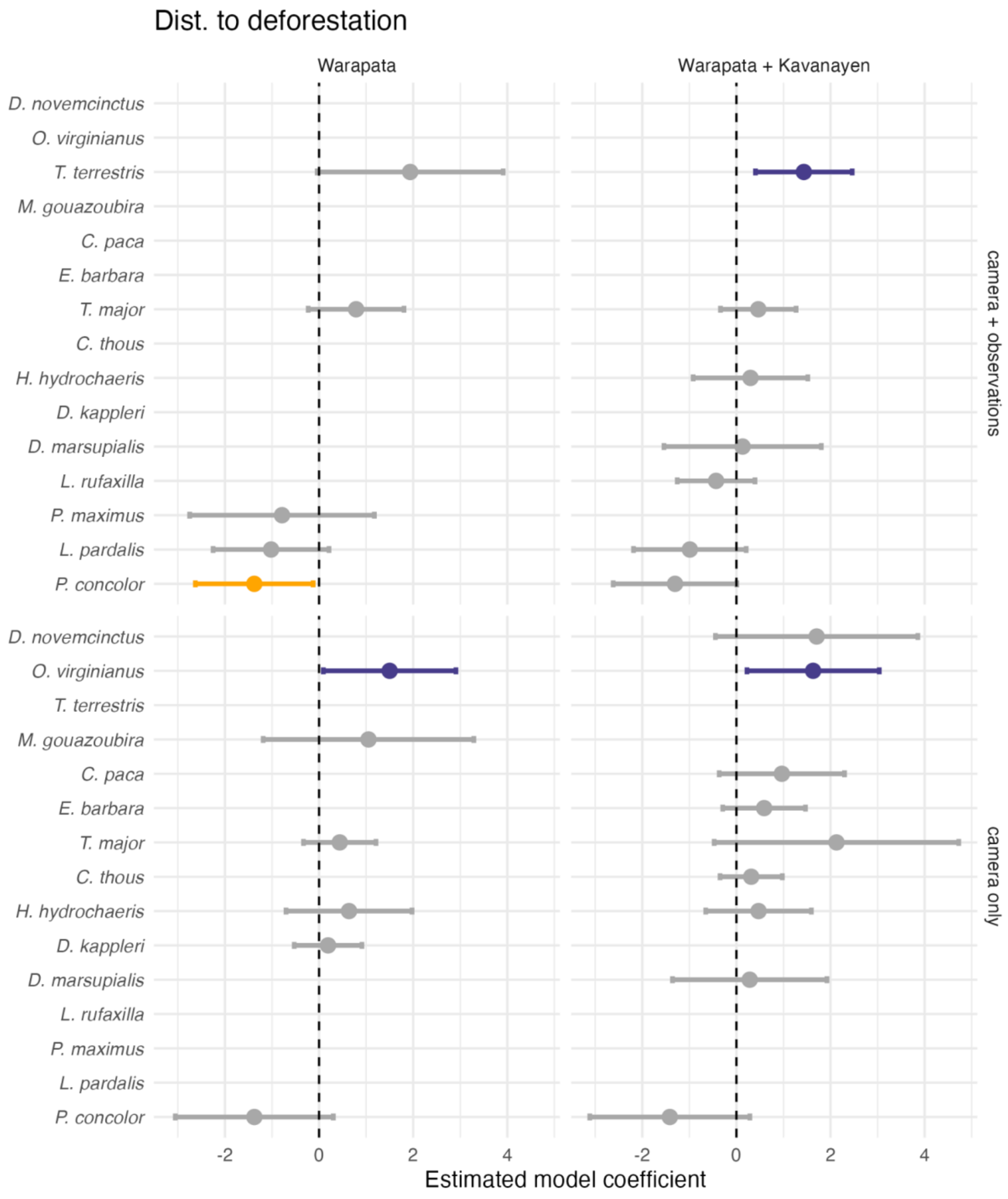
Dist. to deforestation
Warapata
Warapata + Kavanayen
D. novemcinctus
O. virginianus
T. terrestris
M. gouazoubira
C. paca
E. barbara
T. major
C. thous
H. hydrochaeris
D. kappleri
D. marsupialis
L. rufaxilla
P. maximus
L. pardalis
P. concolor
camera + observations
camera only
-2
0
2
4
Estimated model coefficient

c)

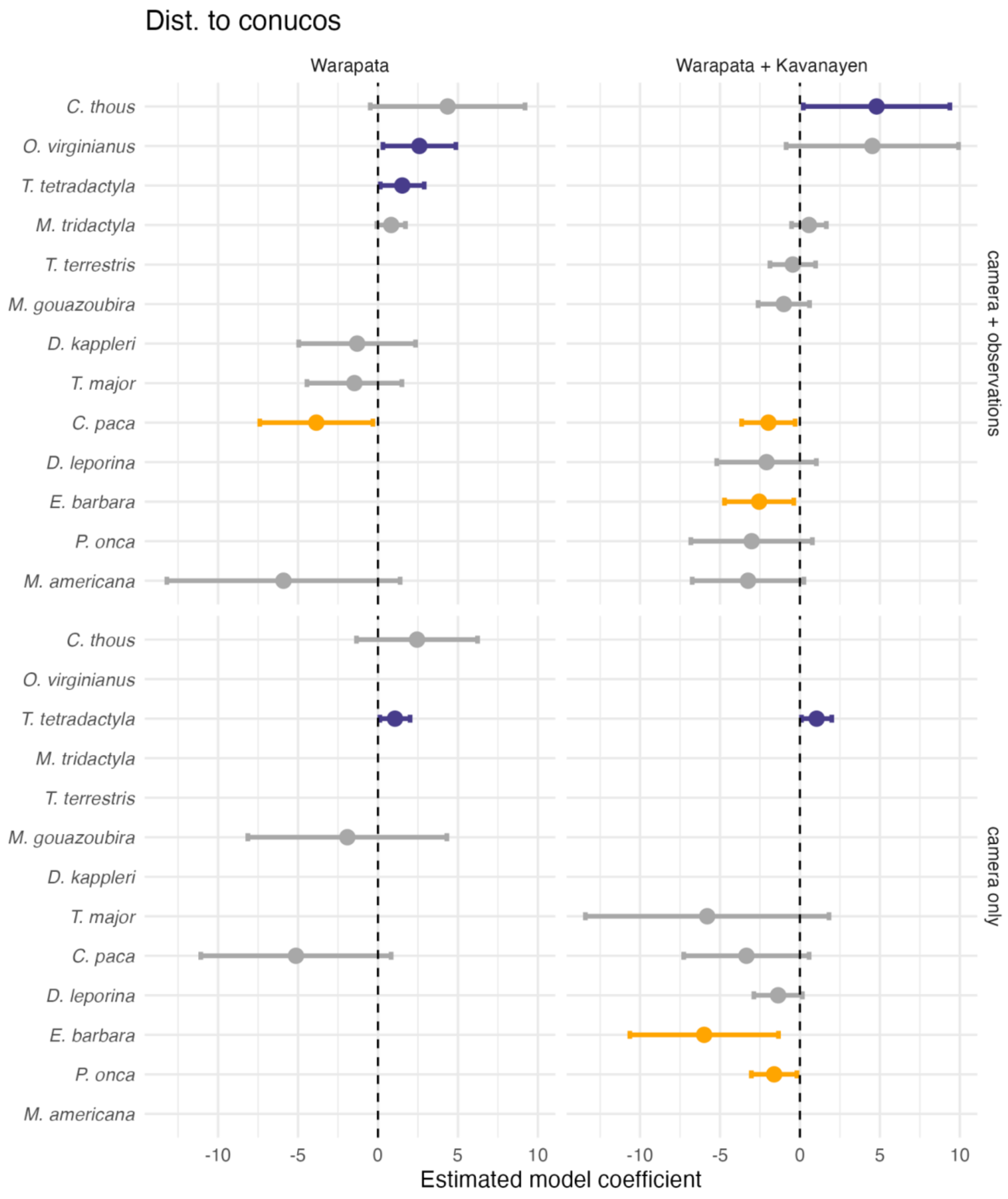

Dist. to conucos
Warapata
Warapata + Kavanayen
C. thous
O. virginianus
T. tetradactyla
M. tridactyla
T. terrestris
M. gouazoubira
D. kappleri
T. major
C. paca
D. leporina
E. barbara
P. onca
M. americana
camera + observations
camera only
-10
-5
0
5
10
Estimated model coefficient

d)

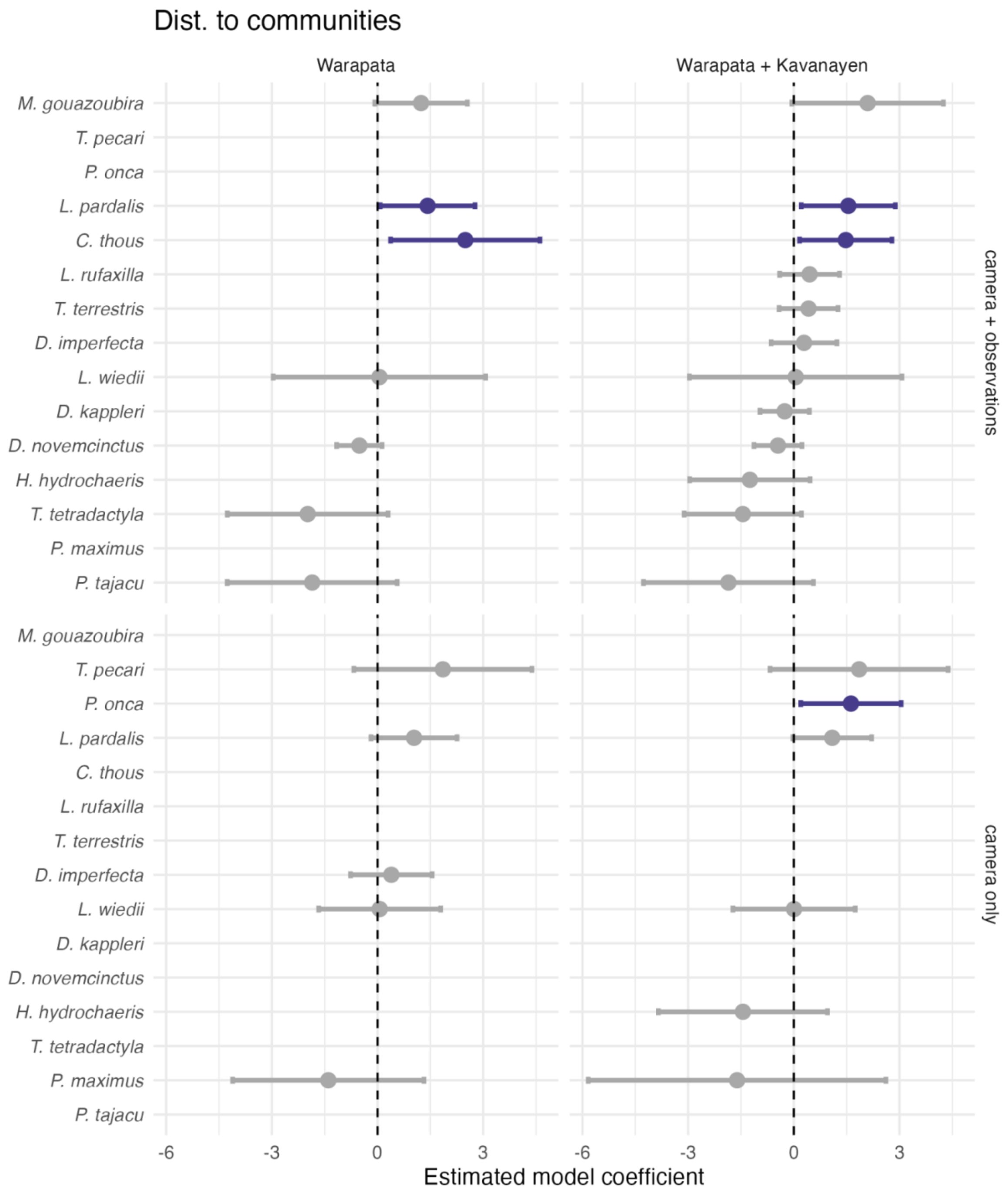
Dist. to communities
Warapata
Warapata + Kavanayen
M. gouazoubira
T. pecari
P. onca
L. pardalis
C. thous
L. rufaxilla
T. terrestris
D. imperfecta
L. wiedii
D. kappleri
D. novemcinctus
H. hydrochaeris
T. tetradactyla
P. maximus
P. tajacu
camera + observations
camera only
-6
-3
0
3
Estimated model coefficient

e)

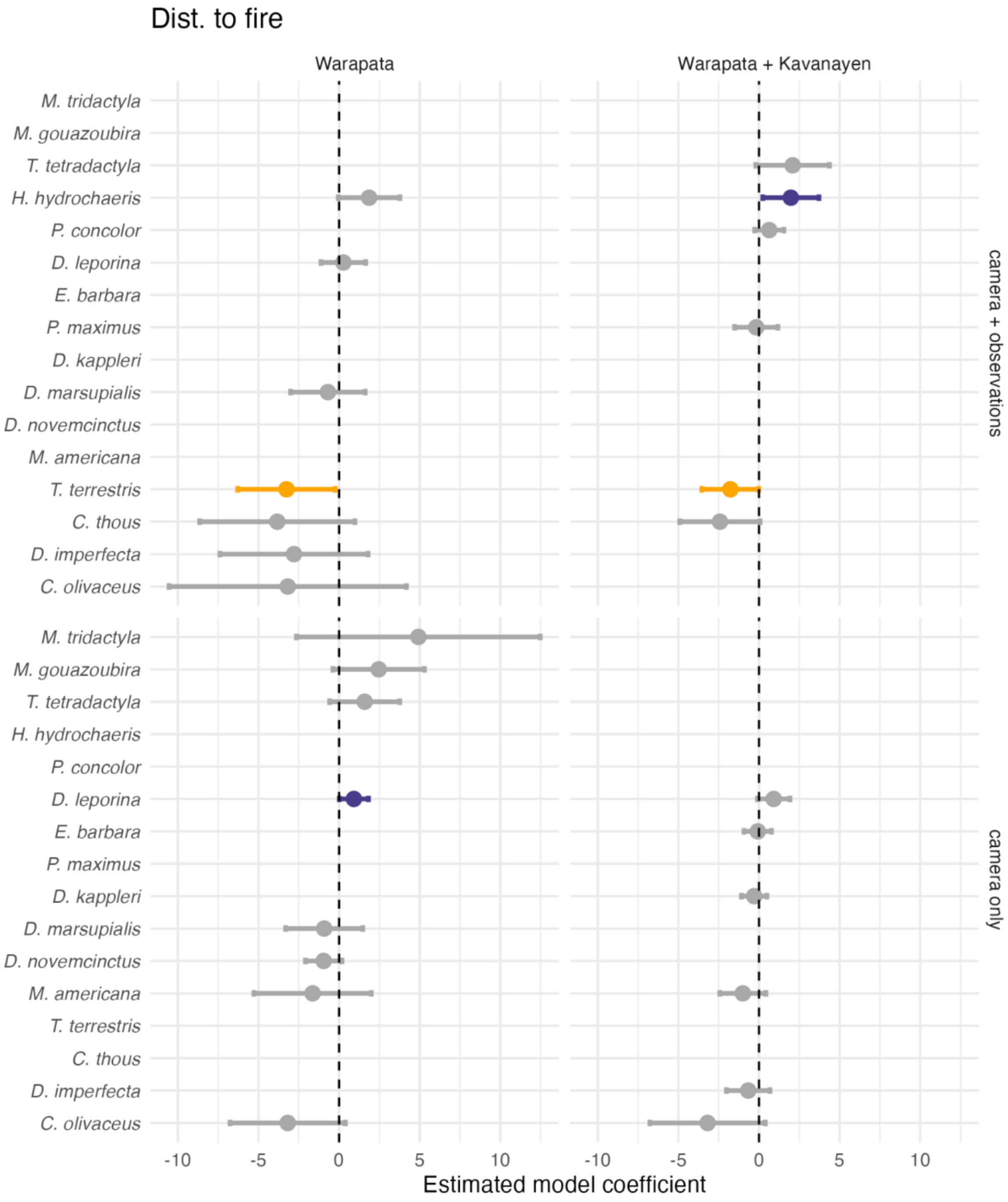

Dist. to fire
Warapata
Warapata + Kavanayen
M. tridactyla
M. gouazoubira
T. tetradactyla
H. hydrochaeris
P. concolor
D. leporina
E. barbara
P. maximus
D. kappleri
D. marsupialis
D. novemcinctus
M. americana
T. terrestris
C. thous
D. imperfecta
C. olivaceus
camera + observations
camera only
-10
-5
0
5
10
Estimated model coefficient

Forests: preferred, disappearing resources

Unsurprisingly, the majority of species had a preference for forest ecosystems (87%), with a significant positive relation registered for red brocket (*M. americana*), lowland paca (*C. paca*), South American tapir ((*T. terrestris*), jaguar (*P. onca*), giant armadillo (*P. maximus*), nine-banded armadillo (*D. novemcinctus*), tayra (*E. barbara*) and greater long-nosed armadillo (*D. kappleri*) (Fig. 5a, Fig. Sup.4_1a, [1]). Those species have been repeatedly reported in forests throughout South America [2–4] and for Pemón [5] and other indigenous groups they are important hunting prey [6]. *T. terrestris* being species that are ecologically related to forest avoided places where deforestation occurred (Fig. 5b, Fig. Sup.4_1b). *T. terrestris* is a cryptic species for which deforestation is one the most important threats. Carnivores species *C. thous* that prefers savanna, avoided communities (Fig. 5d, Fig. Sup.4_1d), which might be explained by the presence of domesticated dogs that are competitors or predators of this omnivore [7].

There was one species, puma (*P. concolor)*, that has strong but not significant positive relation (was attracted) to sites recently deforested (Fig, 5b, Fig. Sup.4_1b). This result corroborates studies on *P. concolor*, possessing high persistence in human-dominated landscapes, showing higher adaptable capability for large carnivores than previously assumed. Pumas in Argentina seek modified landscapes with a greater number of prey species, where the habitat includes presence of bushland and structural complexity. Habitat use and activity patterns of puma in a human-dominated landscape of central Argentina [8] are similar in GS, where they had a higher association with shrub vegetation than forest [1].

Savanna: dominant habitat, poorly investigated fauna

Gran Sabana is characterized by a high diversity of plant communities, distributed in a mosaic pattern, with complex vegetation and environmental transition zones. The current vegetation cover is considered as a transition stage in a long-term savanization process, originally produced by fires and conditioned by the low intrinsic resistance capacity of the forest's nutrient stress and soil, caused by calcium deficiency, aluminum toxicity and drought stress [9].

In this study, one-third of camera traps were located in the savanna for a better understanding of this ecosystem's mammal richness. Four dominant environmental factors that determine savanna structure and function are available moisture and nutrients, fire occurrence, impacts

from herbivory, and the interactions between these environmental determinants [10]. The species with clear association to savanna was *Cerdocyon thous* (Fig 5a, [1], the one with highest detection frequency among all species. *Cerdocyon thous* is present in different types of vegetation from forest to marshland and savanna, but has a preference for savanna [11]. The other species with strong but not significant association to savanna was *Hydrohoreis hydrochaeris*, which was very poorly detected in the study area, but known for its preference for savanna habitat with access to different water bodies [12].

Fire: faunal indifference?

We found that *T. terrestris* is significantly attracted by fire events (Fig. 5e, Fig. Sup.4_1e) that is in contradiction with the knowledge of the forest-specific species [13]. However, a recent study in Brazil Amazon has revealed that tapir can spend more time in degraded forests than in pristine and also defecates and deposits more seeds in degraded areas [14]. We are still missing a clear response of mammals to fire impact on GS, it might be partially attributed to the limitations in the remote sensing data and poor model performance, but it points out to a wide tolerance of fauna to this disturbance regime. The mosaic savanna-forest landscape of Gran Sabana has been present for millennia [15], so the fauna may have adapted to using different habitats, where fire is a constant element of savannas.

Divergent effect of conucos

One of the explanations for forest loss in GS is destabilization of the shifting cultivation system, by extending the cultivation phase, or by reducing the fallow period [16]. In the fertile lands with diabase intrusions, a fallow period of 12 years should be observed, but on the poor soils, which dominate GS, the local inhabitants maintain a 12-year rest period, which should be much longer, calculated between 50 and 100 years. Nevertheless, the deforestation induced by the creation of *conuco* in the study area was at the lowest level of less than one percent during 2015-2019.

Species attracted to *conuco* were *C.paca* and *E.barbara* consistent with *The Garden Hunting hypothesis*, stating that shifting cultivation schemes may generate favorable conditions, increasing the abundance of small and medium wildlife species close to the ‘gardens’ providing game for indigenous hunters [5]. *C.paca* was species potentially hunted by humans, while *E.barbara* can be attracted to *conuco* as small rodents and sometimes also chicken from human settlements represent easy prey for his carnivorous species.

References:

1. Stachowicz I., Ferrer-Paris JR., Quiroga-Carmona M., Moran L., Lozano C. Baseline for monitoring and habitat use of medium to large non- volant mammals in Gran Sabana , Venezuela. Therya. 2020;11: 1–12. doi:10.12933/therya-20-891

2. Francesconi W, Bax V, Blundo-Canto G, Willcock S, Cuadros S, Vanegas M, et al. Hunters and hunting across indigenous and colonist communities at the forest-agriculture interface: An ethnozoological study from the Peruvian Amazon. J Ethnobiol Ethnomed. 2018;14. doi:10.1186/s13002-018-0247-2

3. Roopsind A, Caughlin TT, Sambhu H, Fragoso JMV, Putz FE. Logging and indigenous hunting impacts on persistence of large Neotropical animals. Biotropica. 2017;49: 565–575. doi:10.1111/btp.12446

4. Smith DA. Garden game: Shifting cultivation, indigenous hunting and wildlife ecology in western Panama. Hum Ecol. 2005;33: 505–537. doi:10.1007/s10745-005-5157-Y

5. Stachowicz I, Ferrer-Paris JR, Sanchez-Mercado A. Shifting cultivation and hunting across the savanna-forest mosaic in the Gran Sabana, Venezuela: Facing changes. PeerJ. 2021;9. doi:10.7717/peerj.11612

6. Castellanos HG. La cacería de subsistencia en bosques húmedos del neotrópico sudamericano: un análisis y perspectiva regional. Boletín de Antropología, Universidad de Antioquia2. 2001. pp. 73–87.

7. Vanak AT, Gompper ME. Interference competition at the landscape level: The effect of free-ranging dogs on a native mesocarnivore. J Appl Ecol. 2010;47: 1225–1232. doi:10.1111/j.1365-2664.2010.01870.x

8. Guerisoli MDLM, Caruso N, Luengos Vidal EM, Lucherini M. Habitat use and activity patterns of Puma concolor in a human-dominated landscape of central Argentina. J Mammal. 2019;100: 202–211. doi:10.1093/jmammal/gyz005

9. Bilbao BA, Leal A V., Méndez CL. Indigenous Use of Fire and Forest Loss in Canaima National Park, Venezuela. Assessment of and Tools for Alternative Strategies of Fire

Management in Pemón Indigenous Lands. Hum Ecol. 2010;38: 663–673. doi:10.1007/s10745-010-9344-0

10. Hutley LB, Setterfield SA. Savanna. Encyclopedia of Ecology. 2018. pp. 623–633. doi:10.1016/B978-0-12-409548-9.11148-0

11. Lucherini M. Cerdocyon thous. 2015.

12. Castellanos H, Bertsch C, Veit A, Valeris C, Sarmiento W, Rodríguez F. Cosecha de fauna silvestre y acuática por comunidades Ye´kuana y Sanema del Alto Río Caura. Harvest of wildlife by indigenous ye'kwana and Sanema communities from Alto Caura River. Simposio Investigación y Manejo de Fauna Silvestre en Venezuela en homenaje al Dr Juhani Ojasti. 2008. pp. 132–150.

13. Ferreguetti ÁC, Tomas WM, Bergallo HG. Density, occupancy, and detectability of lowland tapirs, Tapirus terrestris, in Vale Natural Reserve, southeastern Brazil. J Mammal. 2017;98: 114–123. doi:10.1093/jmammal/gyw118

14. Paolucci LN, Pereira RL, Rattis L, Silvério D V., Marques NCS, Macedo MN, et al. Lowland tapirs facilitate seed dispersal in degraded Amazonian forests. Biotropica. 2019;51: 245–252. doi:10.1111/BTP.12627

15. Montoya E, Rull V, Stansell ND, Abbott MB, Nogué S, Bird BW, et al. Forest-savanna-morichal dynamics in relation to fire and human occupation in the southern Gran Sabana (SE Venezuela) during the last millennia. Quat Res. 2011;76: 335–344. doi:10.1016/j.yqres.2011.06.014

16. Fölster H. Local Population Concentrations Breakdown of Traditional Swidden Agriculture. Scientia Guaianae No 5. Caracas, Venezuela; 1995. pp. 65-78.
